# Supplementary material for: Increased Microglial Exosomal miR-124-3p Alleviates Neurodegeneration and Improves Cognitive Outcome after rmTBI
Source: Mol Ther. 2019 Nov 27;28(2):503–22. doi: 10.1016/j.ymthe.2019.11.017 (PMC7001001; doi:10.1016/j.ymthe.2019.11.017)
Supplement: Document S1. Figures S1–S3 [file mmc1.pdf]

YMTHE, Volume *28*

Supplemental Information

Increased Microglial Exosomal miR-124-3p Alleviates Neurodegeneration and Improves Cognitive Outcome after rmTBI

Xintong Ge, Mengtian Guo, Tianpeng Hu, Wenzhu Li, Shan Huang, Zhenyu Yin, Ying Li, Fanglian Chen, Luoyun Zhu, Chunsheng Kang, Rongcai Jiang, Ping Lei, and Jianning Zhang

Supplemental Figures

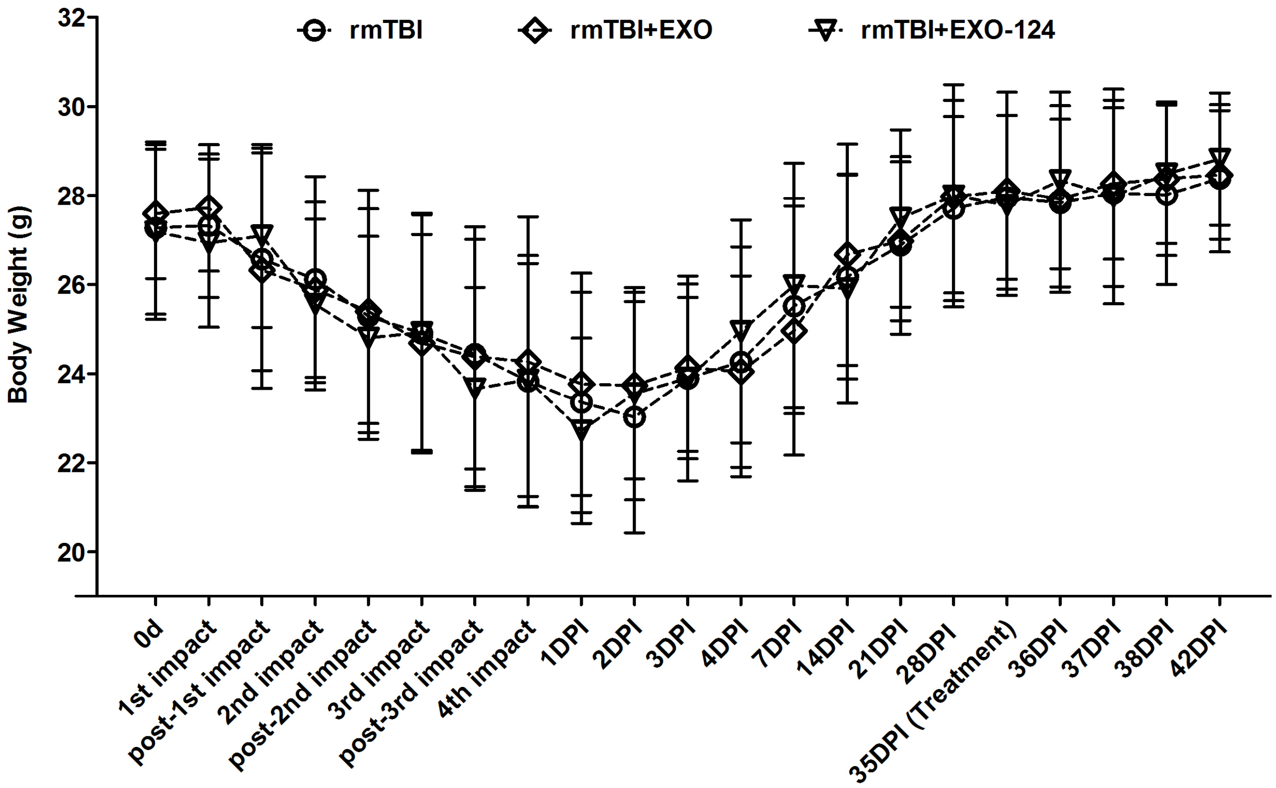

Figure S1. Changes on the body weight of rmTBI mice during the impacting procedure and after injury. The rmTBI mice did loss body weight during the repetitive impacting procedure, but their body weights gradually recovered before receiving treatment at 35 DPI. In addition, no difference was observed among the treatment groups. Abbreviation in the figure: DPI: days post-injury.

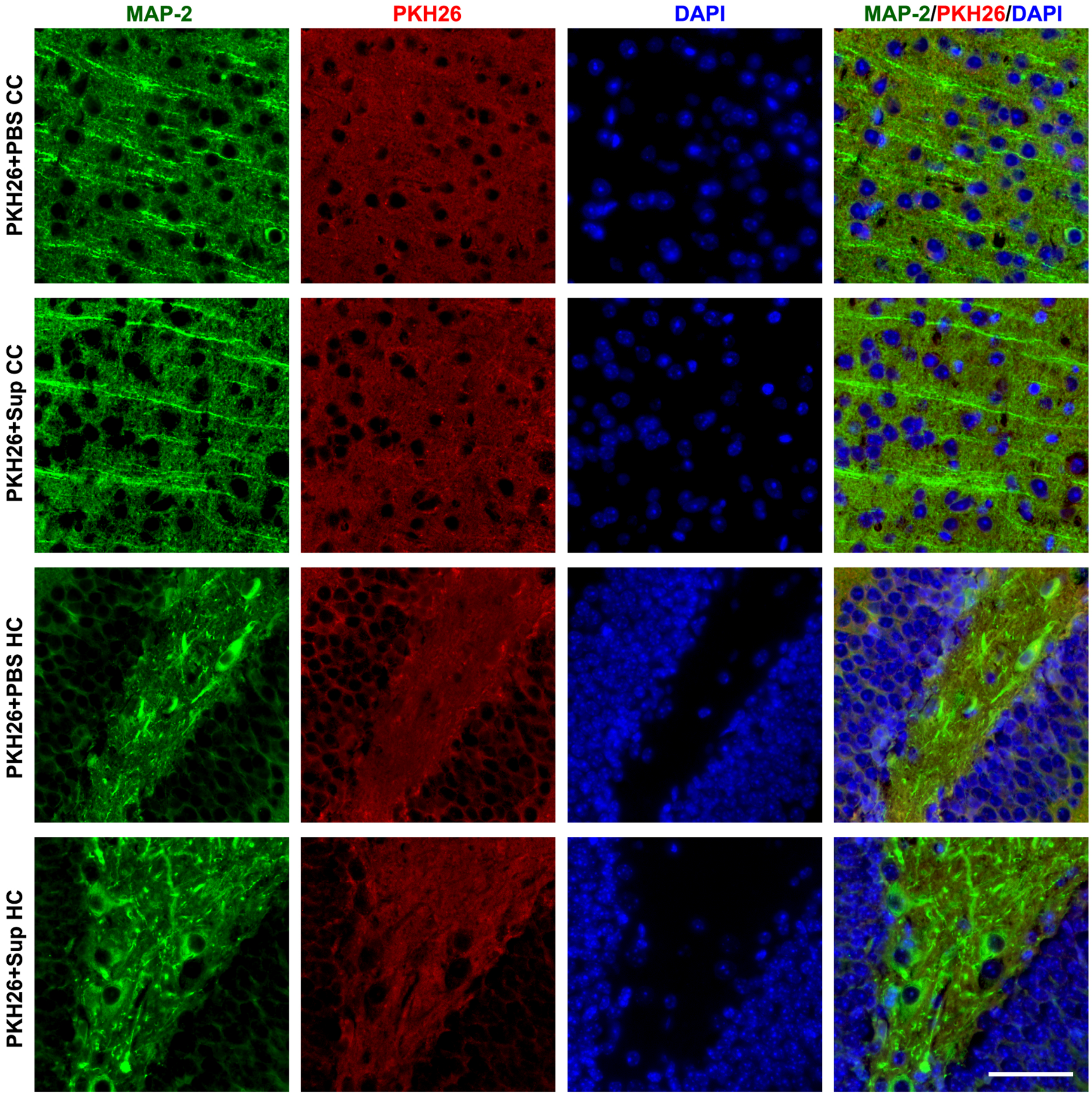

Figure S2. The negative control images of immunofluorescence staining for PKH26 labeled-microglial exosomes in rmTBI mice brain. PKH26 added to PBS and the enriched supernatant (the culture medium of microglia after ultracentrifugation) were injected into rmTBI mice as negative control. Note that PKH26 could be barely observed in the CC and the HC of rmTBI mice at 3 DPI. Scale bars: 50 μm. Abbreviations in the figure: DPI: days post-injury, CC: cerebral cortex, HC: hippocampus.

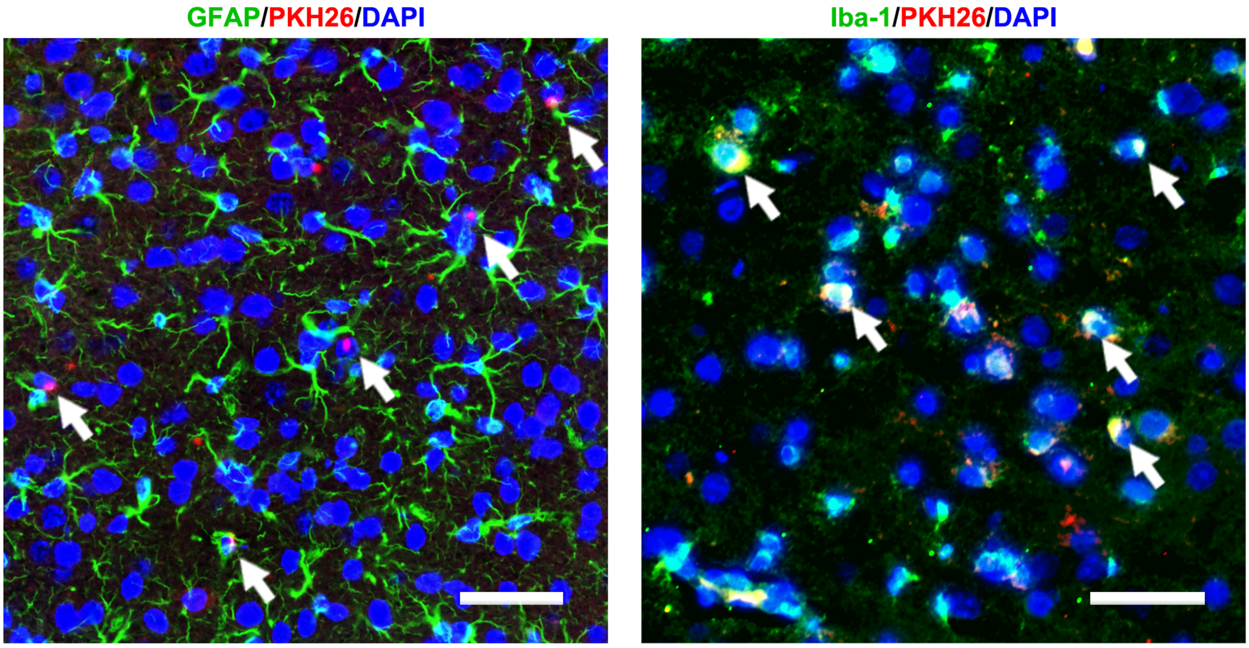

Figure S3. Immunofluorescence staining of astrocytes and microglia in the cerebral cortex of rmTBI mice, taking up exogenous administrated microglial exosomes. The intravenously injected exosomes could be taken up by astrocytes (marked by co-expression of GFAP and PKH26) and microglia (marked by co-expression of Iba-1 and PKH26) at 42 DPI. The double immunostained cells are indicated by white arrows. Note that the number of different types of co-localized neural cells (neurons, astrocytes and microglia) in the pictures has no comparative significance. There is no difference in the number of neurons and astrocytes that co-localized with PKH26 labeled exosomes. Scale bars: 50 μm. Abbreviation in the figure: DPI: days post-injury.
