# Supplementary material for: Increased Microglial Exosomal miR-124-3p Alleviates Neurodegeneration and Improves Cognitive Outcome after rmTBI
Source: Mol Ther. 2019 Nov 27;28(2):503–22. doi: 10.1016/j.ymthe.2019.11.017 (PMC7001001; doi:10.1016/j.ymthe.2019.11.017)
Supplement: Document S2. Article plus Supplemental Information [file mmc2.pdf]

Molecular Therapy
Original Article

Increased Microglial Exosomal miR-124-3p Alleviates Neurodegeneration and Improves Cognitive Outcome after rmTBI

Xintong Ge,[1,2,3] Mengtian Guo,[4,5] Tianpeng Hu,[4,5] Wenzhu Li,[4,5] Shan Huang,[4,5] Zhenyu Yin,[4,5] Ying Li,[2,3] Fanglian Chen,[2,3] Luoyun Zhu,[6] Chunsheng Kang,[2,3] Rongcai Jiang,[1,2,3] Ping Lei,[4,5] and Jianning Zhang[1,2,3]

[1]Department of Neurosurgery, Tianjin Medical University General Hospital, Tianjin 300052, China; [2]Key Laboratory of Injuries, Variations and Regeneration of Nervous System, Tianjin Neurological Institute, Tianjin 300052, China; [3]Key Laboratory of Post-trauma Neuro-repair and Regeneration in Central Nervous System, Tianjin Neurological Institute, Tianjin 300052, China; [4]Laboratory of Neuro-Trauma and Neurodegenerative Disorders, Tianjin Geriatrics Institute, Tianjin 300052, China; [5]Department of Geriatrics, Tianjin Medical University General Hospital, Tianjin 300052, China; [6]Department of Medical Examination, Tianjin Medical University General Hospital, Tianjin 300052, China

Repetitive mild traumatic brain injury (rmTBI) is considered to be an important risk factor for long-term neurodegenerative disorders such as Alzheimer's disease, which is characterized by β-amyloid abnormalities and impaired cognitive function. Microglial exosomes have been reported to be involved in the transportation, distribution, and clearance of β-amyloid in Alzheimer's disease. However, their impacts on the development of neurodegeneration after rmTBI are not yet known. The role of miRNAs in microglial exosomes on regulating post-traumatic neurodegeneration was investigated in the present study. We demonstrated that miR-124-3p level in microglial exosomes from injured brain was significantly altered in the acute, sub-acute, and chronic phases after rmTBI. In *in vitro* experiments, microglial exosomes with upregulated miR-124-3p (EXO-124) alleviated neurodegeneration in repetitive scratch-injured neurons. The effects were exerted by miR-124-3p targeting Rela, an inhibitory transcription factor of ApoE that promotes the β-amyloid proteolytic breakdown, thereby inhibiting β-amyloid abnormalities. In mice with rmTBI, the intravenously injected microglial exosomes were taken up by neurons in injured brain. Besides, miR-124-3p in the exosomes was transferred into hippocampal neurons and alleviated neurodegeneration by targeting the Rela/ApoE signaling pathway. Consequently, EXO-124 treatments improved the cognitive outcome after rmTBI, suggesting a promising therapeutic strategy for future clinical translation.

INTRODUCTION

Traumatic brain injury (TBI) is defined as a head injury suffered from external physical force that induces brain function impairment. It is the most common cause of mortality and disability in children and young adults.[1] More than 50 million people worldwide are affected by a new TBI case annually, with an overall economic cost of about US $406 billion.[2] In addition, as a growing public health problem, TBI is estimated to become the third leading cause of disease burden by 2020.[3]

The degree of brain damage and the symptomology are the criteria to define mild, moderate, and severe TBI. Mild TBI, known as brain concussion, is characterized by a neurological impairment in the absence of structural damage and accounts for almost 70% of TBI cases (upward of 40 million events) reported each year.[4,5] Athletes in full-contact sports, military personnel, and elderly people with declined mobility are at high risk of exposure to repetitive mild TBI (rmTBI).[6,7] It could result in long-term neurodegenerative disorders, including Alzheimer's disease (AD), which is characterized by β-amyloid (Aβ) abnormalities in injured brains and neurocognitive impairments.[8]

The mechanism of Aβ deposition in rmTBI has not been fully elucidated. Some research has indicated that rmTBI could lead to axonal injury that disrupts axonal transport and, thus, induce the accumulation of amyloid precursor protein (APP), β-secretase, and γ-secretase in the axonal bulbs and result in the generation and secretion of Aβ from the swollen bulbs into brain parenchyma.[9,10] In addition, Aβ formation after TBI is suggested to be closely related to vascular dysfunction. Blood-brain barrier disruption, hypo-perfusion, and ischemia after TBI may contribute to Aβ abnormalities through activating β- and γ-secretase.[11] Furthermore, Aβ abnormalities induced

Received 10 July 2019; accepted 21 November 2019;
https://doi.org/10.1016/j.ymthe.2019.11.017.

Correspondence: Rongcai Jiang, Department of Neurosurgery, Tianjin Medical University General Hospital, 154 Anshan Road, Tianjin 300052, China.
E-mail: jiang116216@163.com
Correspondence: Ping Lei, Department of Geriatrics, Tianjin Medical University General Hospital, 154 Anshan Road, Tianjin 300052, China.
E-mail: leiping1974@163.com
Correspondence: Jianning Zhang, Department of Neurosurgery, Tianjin Medical University General Hospital, 154 Anshan Road, Tianjin 300052, China.
E-mail: jnzhang@tmu.edu.cn

This is an open access article under the CC BY license (http://creativecommons.org/licenses/by/4.0/).

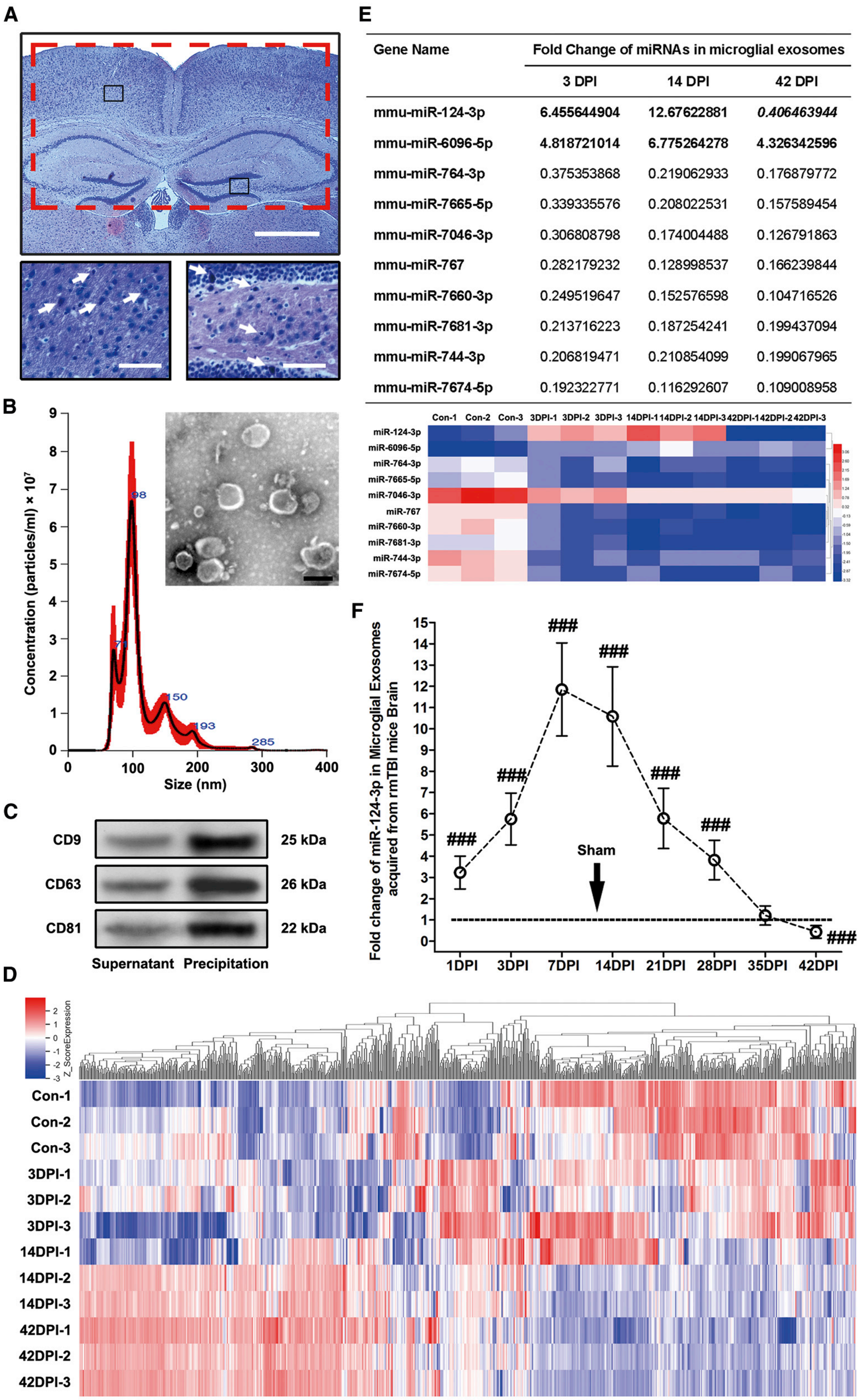

Gene Name	Fold Change of miRNAs in microglial exosomes		
	3 DPI	14 DPI	42 DPI
mmu-miR-124-3p	**6.455644904**	**12.67622881**	***0.406463944***
mmu-miR-6096-5p	**4.818721014**	**6.775264278**	**4.326342596**
mmu-miR-764-3p	0.375353868	0.219062933	0.176879772
mmu-miR-7665-5p	0.339335576	0.208022531	0.157589454
mmu-miR-7046-3p	0.306808798	0.174004488	0.126791863
mmu-miR-767	0.282179232	0.128998537	0.166239844
mmu-miR-7660-3p	0.249519647	0.152576598	0.104716526
mmu-miR-7681-3p	0.213716223	0.187254241	0.199437094
mmu-miR-744-3p	0.206819471	0.210854099	0.199067965
mmu-miR-7674-5p	0.192322771	0.116292607	0.109008958

(legend on next page)

by these acute events after TBI play a significant role in secondary brain injury cascades, including cerebrovascular damage, oxidative stress, and endothelial cell dysfunction. The cascades could be exacerbated after repeated TBI and finally contribute to the development of AD-like pathology and symptoms (post-traumatic neurodegeneration) in later life.[11]

The spread and clearance of Aβ is another important factor that determines the development of Aβ abnormalities and the neurological outcome in neurodegenerative diseases. Serving as the phagocytic cells of the CNS, microglia are the first defensive immune cells of brain and are responsible for Aβ clearance.[12] Recently, growing evidence has shown that the microglial extracellular vesicles (EVs) are involved in the transportation, distribution, and clearance of soluble toxic Aβ peptides in AD.[13] Specifically, the EVs can be divided into exosomes (30–100 nm) and microparticles/microvesicles (100–1,000 nm) by size. They are formed and stored within their origin cells and are released to interact with other cells via a specific receptor at the cell surface or by mixing their cargos with cellular contents after endocytosis. The cargos mainly include proteins and nucleic acids, giving the EVs various types of biochemical potential.[14] For the microglial EVs, previous studies reported that they could carry the soluble neurotoxic Aβ species when the Aβ fibril forms under pathological conditions and activates the innate immune receptor complex located on the cell surface of microglia.[12,15] On the one hand, the microglial EVs containing Aβ may be beneficial, as they could be endocytosed by other microglia and transport Aβ into lysosomes for degradation and clearance.[16] On the other hand, these EVs may also exert detrimental effects by promoting the spread of Aβ to surrounding cells, such as neurons, when the microglia are overloaded with Aβ.[17–19] In addition, the expression of Aβ clearance genes could be downregulated with the progression of AD in response to pro-inflammatory cytokines produced by activated microglia.[20,21] These studies suggest that the beneficial/detrimental effects of microglial exosomes are determined by the progressing stages of AD and are regulated by various factors, such as inflammatory cytokines in the microenvironment of injured brain.

As to rmTBI, the function and underlying mechanism of microglial EVs on regulating Aβ abnormalities and neurodegeneration are not yet known. Clarifying this issue and identifying the factors that determine the impact of microglial EVs in different stages of rmTBI are crucial for exploring therapeutic methods. In our previous studies, we have demonstrated the regulatory effect of M2 microglial exosomes on neuroinflammation after moderate TBI, and we have developed an rmTBI mouse model for further research.[22,23] Based on these studies, we designed this research to investigate the effects of microRNAs (miRNAs) in microglial exosomes on regulating neurodegeneration after rmTBI. The results are expected to expand the previous understanding of the function and mechanism of microglial exosomes in neurodegenerative diseases and open a new avenue of therapeutic strategy for improving the cognitive outcome after rmTBI, using miRNAs in manipulated microglial exosomes.

RESULTS

The miR-124-3p Level in Microglial Exosomes from Injured Brain Is Significantly Altered in Different Stages after rmTBI

To study the roles of microglial exosomal miRNAs in the pathological changes after rmTBI, we developed and used an rmTBI mouse model that produces clinically relevant symptomology, including chronic neurodegeneration and cognitive dysfunction within 5–6 weeks post-injury.[22] The model did not induce macroscopic brain damage and brain edema. However, the eosinophil neurons that indicate neuronal inflammation could be observed in injured brain at 1 DPI (day post-injury), suggesting that the repetitive mild brain injury was successfully established (Figure 1A).[22]

The microglial exosomes were harvested from the mixed tissue of bilateral cerebral cortex and hippocampus (Figure 1A) by ultracentrifugation at 1, 3, 7, 14, 21, 28, 35, and 42 DPI. The exosomes were identified by the transmission electron microscopy (TEM) scanning, nanoparticle tracking analysis (NTA), and western blot. A representative TEM image shows that the precipitated particles were round in shape and within a size range of 30–100 nm, and their peak diameter was further detected to be 96 nm (Figure 1B). Although some precipitated particles were slightly larger than 100 nm in size, the characteristic biomarkers for exosomes, including CD9, CD63, and CD81 were all highly expressed in the precipitation (Figure 1C), indicating that exosomes were the major component of the isolated EVs from rmTBI mouse brains.

A miRNA microarray was then performed to acquire the time profiling of miRNA levels in microglial exosomes after rmTBI

Figure 1. miRNA Microarray Analysis and qRT-PCR Validation for Microglial Exosomes Acquired from Mouse Brains of Different Time Points after rmTBI
(A) Representative H&E staining of rmTBI mouse brains at 1 day post-injury (DPI). Note that the rmTBI model did not induce macroscopic brain damage and brain edema. However, the eosinophil neurons (marked by white arrows in the high-magnification images from the black boxes) that indicate neuronal inflammation could be observed in injured brain, suggesting that the mild brain injury was successfully established. The red box indicates the location of the acquired sample from injured brain, including bilateral cerebral cortex and hippocampus. Scale bars: 500 μm (low magnification) and 100 μm (high magnification). (B) Microglial exosome identification using transmission electron microscopy scanning and nanoparticle tracking analysis. Scale bar: 100 nm. (C) Immunoblotting of exosomal biomarkers, including CD9, CD63, and CD81. (D) Heatmap of the Agilent miRNA microarray that detected the miRNA levels in microglial exosomes after rmTBI. The miRNA microarray contains 1,902 probes for mature miRNA. n = 3 per group. (E) Fold change and heatmap of miRNAs with a level change of more than 2-fold (up- or downregulated) and a p value of less than 0.05 at 3 DPI (acute phase), 14 DPI (sub-acute phase), and 42 DPI (chronic phase) after rmTBI detected by miRNA microarray. miR-124-3p is the only miRNA that upregulated at 3 and 14 DPI and downregulated (reversely expressed) at 42 DPI. Its expression changes after rmTBI are also particularly evident among all miRNAs in microglial exosomes. n = 3 per group. (F) Time profiling of miR-124-3p levels in microglial exosomes after rmTBI determined by qRT-PCR. n = 6 per group. The data are presented as mean ± SD. $^{###}p < 0.001$ versus the sham group.

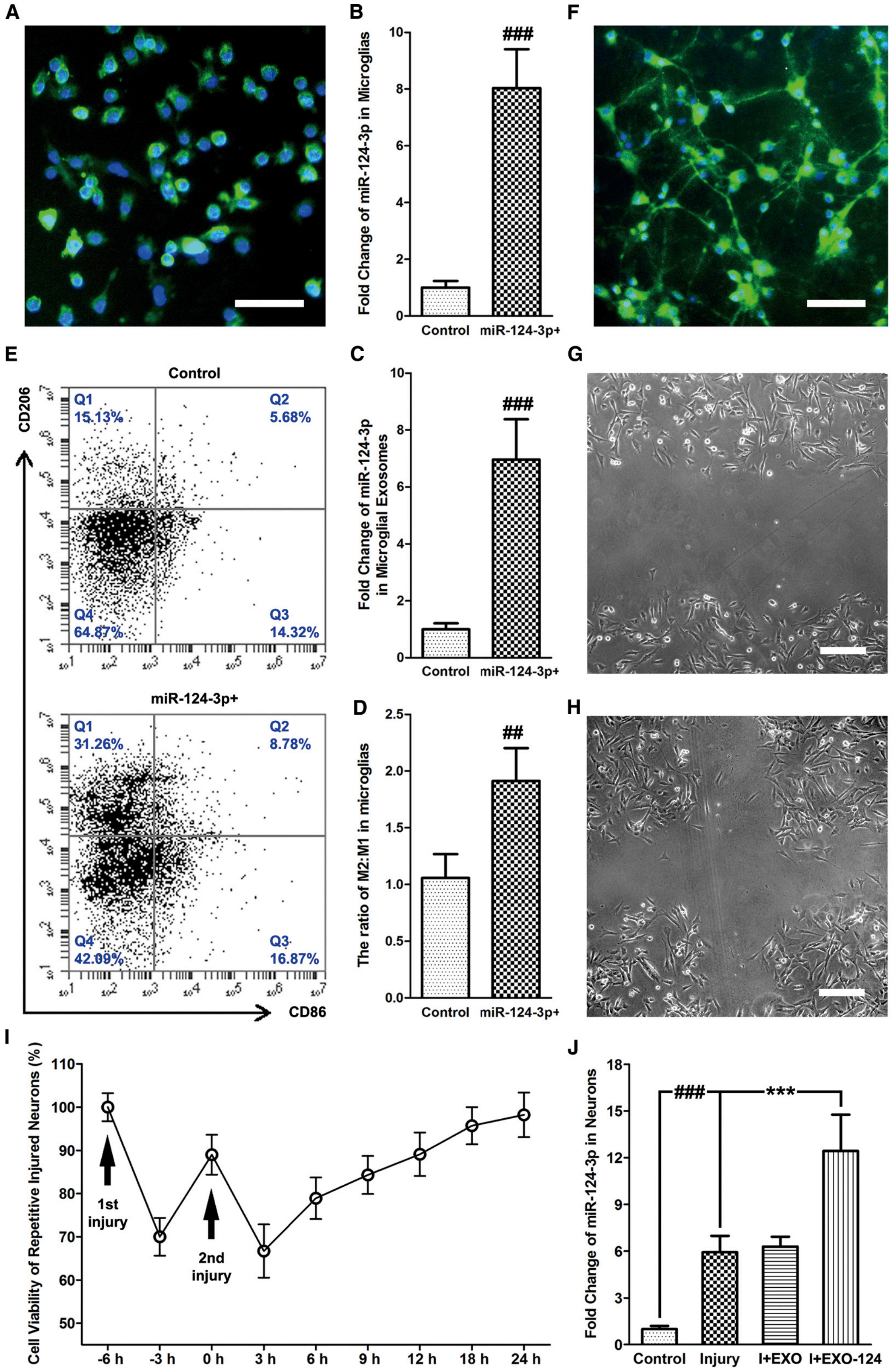

(legend on next page)

(for the heatmap, see Figure 1D; original data have been deposited into the GEO database with the accession number GEO: GSE133997). 3, 14, and 42 DPI were selected to represent, respectively, the time points of acute phase, sub-acute phase, and chronic phase after rmTBI. The results showed that 10 miRNAs had an expression change of more than 2-fold (up- or downregulated), with a p value of less than 0.05 at the 3 time points. Among them, the expression changes of miR-124-3p were particularly evident. Besides, miR-124-3p was the only miRNA that upregulated at 3 and 14 DPI and downregulated (reversely expressed) at 42 DPI, suggesting that its low expression in the chronic phase after rmTBI may exert a reverse impact on neural pathology compared with the acute and sub-acute phases (Figure 1E). Based on these findings, we further verified the expression change of miR-124-3p in microglial exosomes after rmTBI using qRT-PCR. The miR-124-3p level was increased since 1 DPI, reached the peak at 7–14 DPI, then gradually decreased to the baseline at 35 DPI, and finally dropped down to almost half below the baseline at 42 DPI (Figure 1F). These results were consistent with those of our previous findings with repeated moderate TBI.[23] In addition, microglial exosomes have been proved to be effective in attenuating ischemic brain injury and promoting neuronal survival via miR-124-3p modulation.[24] Therefore, we inferred that microglial exosomal miR-124-3p can be a significant factor regulating the pathological development after rmTBI.

Upregulated miR-124-3p in Microglia Promotes M2 Polarization *In Vitro*

To investigate the function of microglial exosomal miR-124-3p on neurodegeneration, miR-124-3p mimics were transfected into a cultured BV2 mouse microglia cell line to acquire microglial exosomes with upregulated miR-124-3p (EXO-124). We identified the cultured microglia by immunofluorescence staining of Iba-1 (Figure 2A) and confirmed that the transfection could increase miR-124-3p levels in both the cells and their exosomes (Figures 2B and 2C).

Macrophages and microglia are generally divided into two types: classically activated (M1) and alternatively activated (M2). In most pathological conditions, the M1 microglia is pro-inflammatory, but the M2 microglia is anti-inflammatory and neuroprotective. However, some researchers have argued that the concept of M2 microglia may expand the scope of initially defined interleukin (IL)-4- and IL-13-activated immunosuppressive microglia and lead to confusion in understanding their functions.[25] In addition, M1 and M2 microglia were also suggested to be only used as an *in vitro* classification.[26] From this point of view, we defined the M1 and M2 subsets in the present study as *in vitro* pro-inflammatory and anti-inflammatory microglia and selected the widely reported CD206 and CD86 to be the biomarkers for M2 and M1 microglia, respectively. The impact of upregulated miR-124-3p on microglial polarization that determines the subsets of microglia was detected by flow cytometry. We found that the ratio between M2 and M1 microglia was increased after miR-124-3p mimic transfection into cultured resting microglia (M0) (Figures 2D and 2E). Therefore, upregulated miR-124-3p in microglia could promote the anti-inflammatory M2 polarization *in vitro* and may exert a beneficial effect on neighbor cells via their transferring by exosomes.

Microglial Exosomal miR-124-3p Alleviates Neurodegeneration in Repetitively Injured Neurons

To study the impact of microglial exosomal miR-124-3p on neurodegeneration after rmTBI *in vitro*, we cultured an HT22 mouse hippocampal neuronal cell line identified by immunofluorescence staining of microtubule-associated protein 2 (MAP-2) (Figure 2F), and performed the repetitive scratch cell injury model. During the procedure, the scratch wound could be observed clearly after each time of injury (Figures 2G and 2H). In addition, the CCK8 assay was performed to detect the impact of repetitive scratch injury on the cell viability of neurons. Utilizing highly water-soluble tetrazolium salt, CCK8 allows convenient colorimetric assays determining the viable cell number with higher sensitivity than that of the 3-(4,5-dimethylthiazol-2-yl)-2,5-diphenyltetrazolium bromide (MTT) assay. The results suggested that the cell proliferation was sharply decreased after the two injuries and gradually recovered to over 90% in 24 h post-injury, indicating that the repetitive cell injury model was successfully established (Figure 2I). Furthermore, the injured neurons were treated with unedited microglial exosomes or EXO-124. We found that the miR-124-3p level in neurons was increased after injury and could be further upregulated by treating with EXO-124. Besides, no expression change was observed in the injured neurons treated with unedited microglial exosomes (Figure 2J)

The development of neurodegeneration in repetitively injured neurons after exosome treatment was evaluated by three aspects. (1) To assess neurite outgrowth of injured neurons, the neurite branches were labeled with the neuron-specific beta III-tubulin by immunofluorescence staining (Figure 3A). We quantified the neurite branch numbers and total neurite length under a microscope and found that the repetitive injury induced a decrease of the branch numbers and neurite length, while EXO-124 treatment reversed the change

Figure 2. Upregulated miR-124-3p in Microglia Promotes M2 Polarization *In Vitro*, and EXO-124 Treatment Increases miR-124-3p Level in Neurons after Repetitive Scratch Injury

(A) Immunofluorescence staining of Iba-1 for identifying cultured microglia. Scale bar: 100 μm. (B and C) The altered miR-124-3p level in (B) microglia and (C) their exosomes after miR-124-3p mimic transfection. (D) Quantitative data for microglia/macrophage subsets in cultured microglia after miR-124-3p mimic transfection. (E) Representative dot spot of flow cytometry for microglia/macrophage subsets in cultured microglia after miR-124-3p mimic transfection. CD206 and CD86 are the selected biomarkers of M2 and M1 microglia, respectively. (F) Immunofluorescence staining of MAP-2 for identifying cultured neurons. Scale bar: 100 μm. (G and H) Cultured neurons after (G) first scratch and (H) second scratch under transmission light microscope. Scale bars: 50 μm. (I) Cell viability of repetitive scratch-injured neurons. (J) The altered miR-124-3p level in neurons after injury and EXO-124 treatment. n = 6 per group. The data are presented as mean ± SD. ***$p < 0.001$ versus the I+EXO-124 group. $^{\#\#}p < 0.01$ and $^{\#\#\#}p < 0.001$ versus the control group.

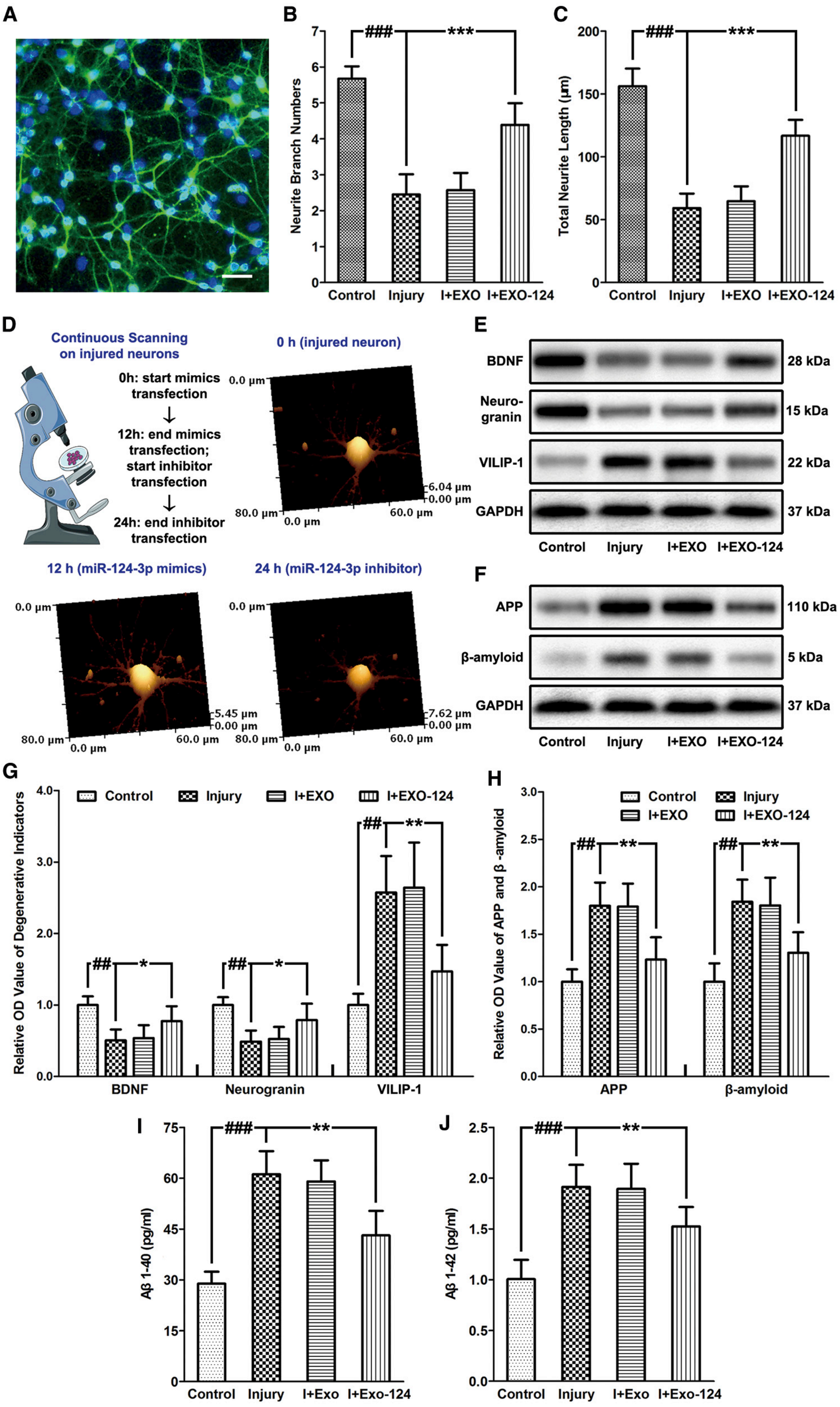

(legend on next page)

(Figures 3B and 3C). In addition, hopping probe ion conductance microscopy (HPICM) continuous topological scanning was performed to further observe the microscopic changes in injured neurons after successive transfection of miR-124-3p mimics and miR-124-3p inhibitor. It suggested that the branch numbers and neurite length of injured neurons were restored by transfection with miR-124-3p mimics for 12 h but decreased after transfection with miR-124-3p inhibitor for another 12 h (Figure 3D). From this, we could draw the conclusion that EXO-124 treatment contributed to promoting neurite outgrowth after cell injury via transferring miR-124-3p into neurons. (2) To detect the expression of neuroprotective factors and neurodegenerative indicators, the expression levels of brain-derived neurotrophic factor (BDNF), neurogranin, and VILIP-1 in neurons after injury and exosome treatment were quantified. BDNF is a member of the neurotrophin family, which contributes to supporting the survival of existing neurons, encouraging the growth and differentiation of newborn neurons, and enhancing the synaptic plasticity. It is a classic neuroprotective factor, as its disruption is considered to be a significant risk factor for AD and age-related cognitive decline.[27] Neurogranin is a calmodulin-binding protein primarily expressed in dendritic spines. It exerts a neuroprotective effect in the pathological development of cerebral ischemia.[28] As a postsynaptic protein, it also acts as an indicator of mild TBI[29] and AD.[30,31] VILIP-1 is a neuron-specific calcium sensor protein that modulates intracellular signaling pathways by directly or indirectly regulating the activity of adenylyl cyclase. It has been identified to be a potential neurodegenerative biomarker with an elevated level in ischemic stroke, TBI, and early-stage AD patients.[32–34] Western blot results have shown that the repetitive injury induced a decreased expression of BDNF and neurogranin and an increased expression of VILIP-1 in neurons. In addition, these expression changes were reversed by EXO-124 treatment (Figures 3E and 3G), suggesting that the neurodegeneration led by repetitive injury could be inhibited by microglial exosomal miR-124-3p. (3) The Aβ abnormalities were evaluated in injured neurons after exosome treatment. Aβ is the main component of the amyloid plaques (senile plaques) that are found in the brain of AD patients. Its deposition also occurs at an early age and at an accelerated rate in rmTBI patients, which is associated with increased clinical and pathological severity.[35] In addition, Aβ is derived from its precursor protein, APP, and aggregates to form soluble oligomers $A\beta_{1-40}$ and $A\beta_{1-42}$, which are toxic to nerve cells.[36] Therefore, the expression levels of APP and Aβ in neurons, and those of $A\beta_{1-40}$ and $A\beta_{1-42}$ in the culture medium, were assessed. We found that all of their expressions were promoted after injury and were suppressed after EXO-124 treatment (Figures 3F and 3H–3J), indicating that the Aβ abnormalities in neurons induced by repetitive injury can be inhibited by microglial exosomal miR-124-3p. Taken together, these results suggested that microglial exosomal miR-124-3p could alleviate neurodegeneration in repetitive injured neurons via their transfer by exosomes.

Microglial Exosomal miR-124-3p Alleviates Neurodegeneration by Targeting the Rela/ApoE Signaling Pathway in Repetitively Injured Neurons

To explore the mechanisms underlying the effects of miR-124-3p on neurodegeneration, we first predicted the potential target genes of miR-124-3p and picked out 22 genes related to the process of neurodegeneration or amyloid deposition using the OMIM database. A genetic interaction network of miR-124-3p with these genes was built, and we found that Rela is one of the core genes connected with other genes in the network (Figure 4A). In addition, KEGG analysis for the predicted miR-124-3p targeted genes was performed. It indicated that Rela is the only gene that was involved in all the top three enriched signaling pathways (the neurotrophin signaling pathway, focal adhesion, and cyclic AMP [cAMP] signaling pathway) regulated by the 22 neurodegeneration-associated miR-124-3p targeted genes (Figure 4B). Furthermore, Rela has been widely reported to be involved in regulating neuroinflammation in various pathological conditions.[9] As neuroinflammation is a characteristic pathological change and an important cause of neurodegeneration after rmTBI, we focused our interest on studying the roles of Rela under the regulation of miR-124-3p in the following experiments.

The expression level of Rela in neurons after injury and exosome treatment was detected. We found that its expression was promoted in injured neurons and was suppressed after EXO-124 treatment, suggesting that miR-124-3p could inhibit Rela expression in injured neurons (Figures 5A and 5B). After that, we acquired the potential binding sites in miR-124-3p for Rela 3′ UTR (Figure 5C) and performed the luciferase reporter assay by co-transfecting cultured neurons with the Rela 3′ UTR constructs containing the putative miR-124-3p binding sites with either miR-124-3p mimics or scrambled oligonucleotides. We found that miR-124-3p inhibited the luciferase activity of the wild type (WT), but not the mutant type (Mut), of 3′ UTR reporter constructs (Figure 5D). It indicated that miR-124-3p could directly target Rela and downregulate its expression by binding to the 3′ UTR sites in neurons.

Figure 3. Microglial Exosomal miR-124-3p Alleviates Neurodegeneration in Repetitive Injured Neurons

(A) Representative image of neurite branches detected by immunofluorescence staining of neuronal-specific beta III-tubulin. Scale bar: 100 μm. (B) The neurite branch numbers after repetitive cell injury and exosome treatment. (C) Total neurite length after repetitive cell injury and exosome treatment. (D) The schematic representation and the major findings of the HPICM topological continuous scanning in injured neurons. The neurite branch numbers and total neurite length of neurons could be restored by transfection with miR-124-3p mimics for 12 h but decreased after transfection with miR-124-3p inhibitor for another 12 h. (E) Immunoblotting of neuroprotective factors and neurodegenerative indicators, including BDNF, neurogranin, and VILIP-1 in neurons after injury and exosome treatment. (F) Immunoblotting of APP and Aβ in neurons after injury and exosome treatment. (G) Quantitative data of neuroprotective factors and neurodegenerative indicators, including BDNF, neurogranin, and VILIP-1 in neurons after injury and exosome treatment. (H) Quantitative data of APP and Aβ in neurons after injury and exosome treatment. (I and J) The expression levels of soluble (I) $A\beta_{1-40}$ and (J) $A\beta_{1-42}$ in neuronal culture medium. n = 6 per group. The data are presented as mean ± SD. **p < 0.01 and ***p < 0.001 versus the I+EXO-124 group. ##p < 0.01 and ###p < 0.001 versus the control group.

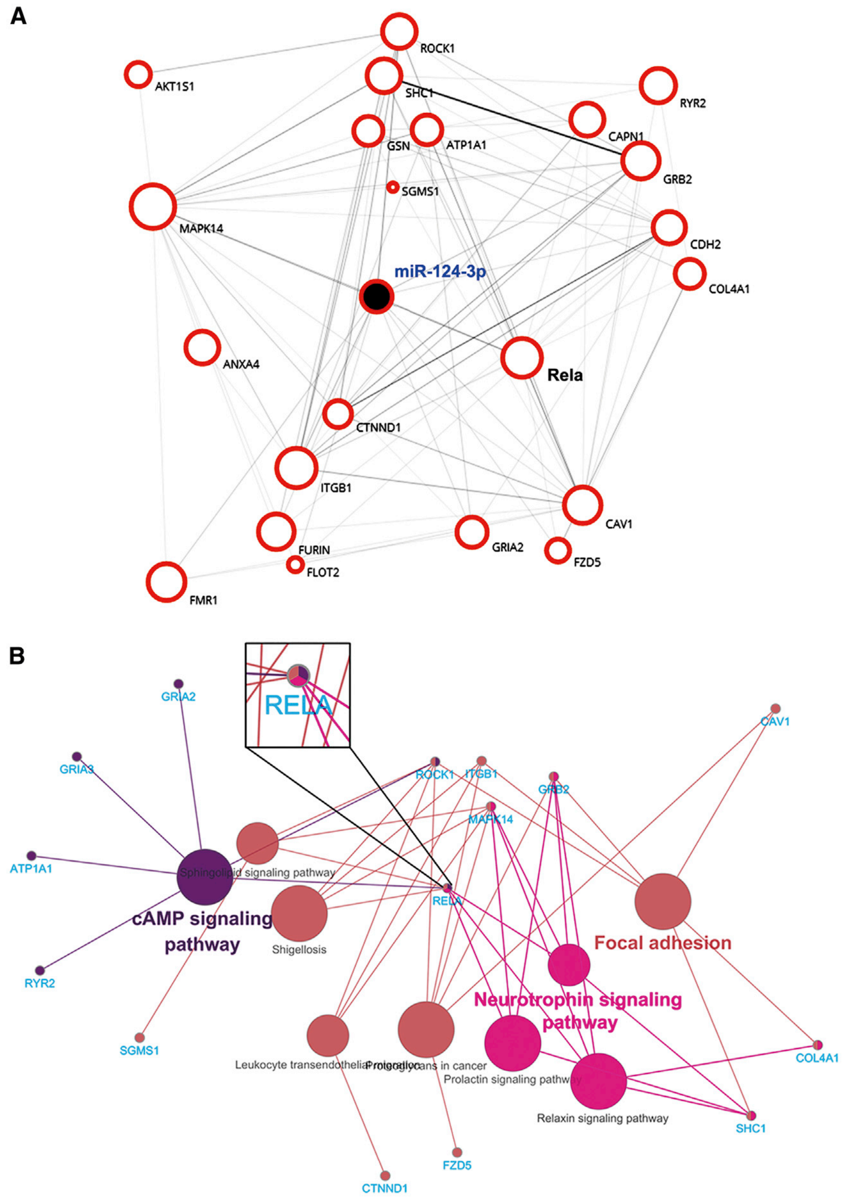

Figure 4. Bioinformatics Analysis of miR-124-3p and Its Downstream Targeted Genes

(A) The genetic interaction network of miR-124-3p built by Biovista Vizit. There are 22 possible targeted genes of miR-124-3p associated with neurodegeneration and amyloid deposition. Rela is one of the core genes connected with other genes in the network. (B) KEGG analysis for the predicted miR-124-3p targeted genes using Cytoscape software. Note that Rela is the only gene that was involved in all the top three enriched signaling pathways (neurotrophin signaling pathway, focal adhesion, and cAMP signaling pathway) regulated by the 22 neurodegeneration-associated miR-124-3p targeted genes.

The target genes of Rela were predicted using an online chromatin immunoprecipitation sequencing (ChIP-seq) database, and those that were related to the process of neurodegeneration or amyloid deposition were further picked out. A qPCR microarray was then performed to detect the mRNA expression levels of these neurodegeneration-related genes that are potentially targeted by Rela under the regulation of miR-124-3p in injured neurons. The results suggested that the expression of ApoE was altered most apparently among the 26 genes by miR-124-3p mimics (Table 1). As ApoE is a well-known gene that could enhance the proteolytic breakdown of Aβ in AD,[37] we inferred that ApoE could regulate Aβ abnormalities in repetitively injured neurons under the regulation of miR-124-3p and Rela.

To verify this hypothesis and clarify the mechanism of exosomal miR-124-3p on alleviating neurodegeneration, we first detected the level change of ApoE in neurons after injury and exosome treatment. We found that the expression of ApoE was suppressed after injury and could be promoted by EXO-124 treatment (Figures 5E and 5F). In addition, the expression level of ApoE in Rela-overexpressed (Rela^{+}) neurons after injury and EXO-124 treatment was also detected. Compared with the unedited neurons, the Rela^{+} neurons showed a decreased expression of ApoE, indicating that overexpression of Rela in injured neurons could block the promoting effect of miR-124-3p on ApoE expression. Besides, no difference in ApoE level was observed between the unedited injured neurons and the Rela^{+} injured neurons treated with EXO-124. It further suggested that exosomal miR-124-3p promoted ApoE expression mainly through targeting the Rela/ApoE signaling pathway in injured neurons (Figures 5G and 5H).

Based on these findings, we got the ApoE promoter sequences from the ENCODES project (Figure 5I) and performed the luciferase reporter assay by co-transfecting cultured neurons with the ApoE promoter reporter constructs and a Rela expression vector or an empty vector. We found that the WT, but not the Mut, ApoE reporter constructs showed a significant decrease of the luciferase activity when co-transfected with Rela expression vector, suggesting that Rela could inhibit the promoter activity of ApoE in neurons (Figure 5J). Then, we performed the ChIP-PCR assay, in which significant enrichment of Rela in the first intron of the ApoE gene was observed in the neurons incubated with anti-FLAG tag or Rela antibody (Figure 5K). In addition, the sequences of the promoter-flanking region of ApoE from mouse, human, and rat were aligned, which showed obviously conserved nucleotides and consensus binding sites for Rela (Figure 5L). Taken together, these results suggested that Rela could downregulate ApoE expression by targeting its

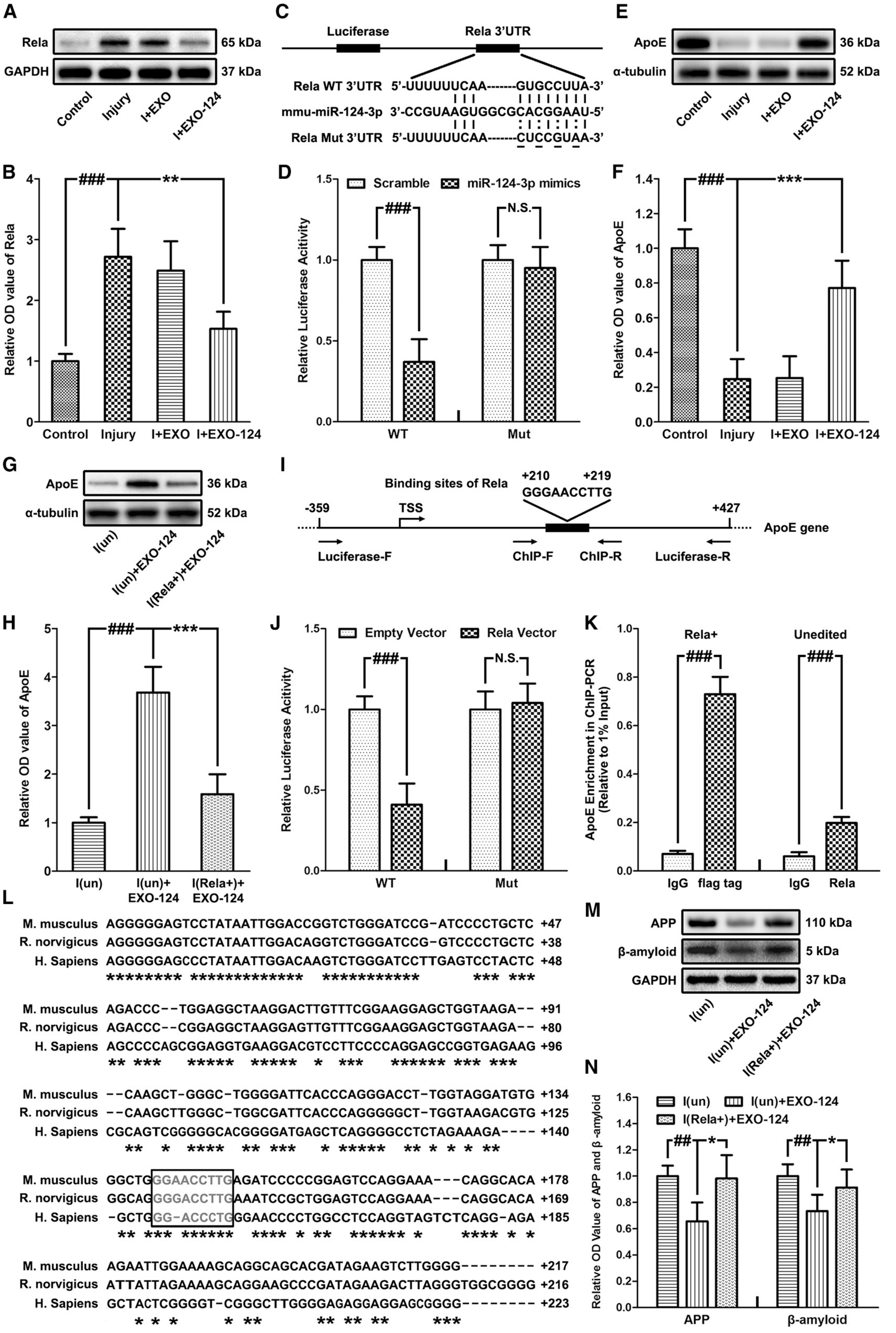

(legend on next page)

binding sites in the first intron of ApoE as an inhibitory transcription factor in neurons.

To further validate the regulative effect of miR-124-3p on neurodegeneration through the Rela/ApoE signaling pathway, we also detected the expression levels of APP and Aβ in unedited and $Rela^+$ neurons after injury and EXO-124 treatment. We found that their expression could be suppressed by EXO-124 treatment in unedited injured neurons but not in the $Rela^+$ injured neurons (Figures 5M and 5N). Consequently, we could draw the conclusion that the inhibitory effect of microglial exosomal miR-124-3p on Aβ abnormalities and neurodegeneration in neurons was exerted by targeting the Rela/ApoE signaling pathway.

Microglial Exosomes with Upregulated miR-124-3p Contribute to Alleviating Neurodegeneration in rmTBI Mice by Targeting the Rela/ApoE Signaling Pathway in Hippocampal Neurons

To explore the therapeutic effects of EXO-124 on neurodegeneration in rmTBI mice and further confirm the mechanism of microglial exosomal miR-124-3p *in vivo*, we treated rmTBI mice with PKH26-labeled EXO-124 at 35 DPI, when the endogenous microglial exosomal miR-124-3p level decreased to the baseline after injury. Specifically, the exosomes were intravenously injected via tail vein at a dose of 3×10^{10} in 200 μL PBS per mouse.[38]

Although the mice did lose body weight during the repetitive impacting procedure, they rapidly regained spontaneous respiration, righting reflex, and the ability to ambulate after each time of injury. The body weight gradually recovered before receiving treatment at 35 DPI, and no difference was observed among the treatment groups after injury (Figure S1). The immunofluorescence staining of injured brain at 42 DPI showed that MAP-2 and PKH26 were co-expressed in the neurons of cerebral cortex and hippocampus, indicating that the intravenously injected exosomes could be taken up by neurons (Figure 6A; Figure S2). Besides, the glial fibrillary acidic protein (GFAP) (biomarker of astrocytes) and PKH26 double-immunostained cells, as well as the Iba-1 and PKH26 double-immunostained cells, were observed in injured brain, suggesting that the exosomes were also taken up by astrocytes and microglia (Figure S3). Furthermore, the miR-124-3p level in the cerebral cortex and hippocampus of rmTBI mice at 42 DPI was increased significantly after EXO-124 treatment (Figure 6B). These results suggested that the exogenous exosomal miR-124-3p had a satisfied *in vivo* transfer efficacy in the chronic phase of rmTBI.

Based on these results, the expression levels of Rela, ApoE, APP, Aβ, $A\beta_{1-40}$, and $A\beta_{1-42}$ were determined in the hippocampus of rmTBI mice. We found that the expression of Rela was promoted in rmTBI mice and was inhibited after EXO-124 treatment. Besides, the decreased expression of ApoE in rmTBI mice was restored after EXO-124 treatment (Figures 6C and 6D). For Aβ abnormality measurement, the expression levels of APP, Aβ, $A\beta_{1-40}$, and $A\beta_{1-42}$ were all increased in rmTBI mice and can be decreased by EXO-124 treatment (Figures 6C and 6E–6G). These results were consistent with the *in vitro* experiments, suggesting that microglial exosomal miR-124-3p could alleviate neurodegeneration in rmTBI mice by targeting the Rela/ApoE signaling pathway in hippocampal neurons.

Microglial Exosomes with Upregulated miR-124-3p Improve the Cognitive Outcome after rmTBI

The cognitive outcome of rmTBI mice was evaluated by using the Morris water maze (MWM) test and the novel object recognition test. In the MWM test, the spatial acquisition trials were performed during 43–46 DPI to test the spatial learning ability. Escape latency, which represents the capability to navigate from a starting location to a submerged platform, was gradually decreased in the testing procedure, indicating that a spatial memory was established ($F = 709.31$, $p < 0.001$) (Figure 7A). The probe trials were conducted at 47 DPI to test the retrograde reference memory, in which more time spent in the goal quadrant indicates better memory. Compared with the sham mice, the rmTBI mice showed prolonged escape latency in the spatial acquisition trials and decreased time spent in the goal quadrant in the probe trials. Besides, EXO-124 treatment could improve the impaired performance, manifested by shortening the escape latency ($F = 10.62$, $p = 0.0057$) and increasing the goal quadrant time (Figure 7B). As no difference was observed in swimming speed among the groups, the different performances of mice were not due to motor impairments

Figure 5. Microglial Exosomal miR-124-3p Inhibits Neurodegeneration by Targeting Rela, an Inhibitory Transcription Factor of ApoE, thus Promoting ApoE Expression and Inhibiting Aβ Abnormalities

(A and B) Immunoblotting (A) and quantitative data (B) of Rela in neurons after injury and exosome treatment. (C) Schematic representation of the putative binding sites for miR-124-3p in the Rela 3′ UTR. The wild-type (Rela WT 3′ UTR) and mutant type (Rela Mut 3′ UTR) of luciferase reporter constructs have, respectively, intact and mutated seed sequences (underlined) in the miR-124-3p binding sites. (D) The relative luciferase activity of the Rela 3′ UTR reporter constructs, which were co-transfected with either miR-124-3p mimics or scrambled oligonucleotides into neurons. The data are presented as the ratio of luciferase activity from scrambled oligonucleotides versus miR-124-3p mimic-transfected neurons. (E and F) Immunoblotting (E) and quantitative data (F) of ApoE in neurons. (G and H) Immunoblotting (G) and quantitative data (H) of ApoE in unedited/$Rela^+$ neurons after injury and exosome treatment. (I) Location of the binding sequences of Rela in mouse ApoE gene. (J) The relative luciferase activity of the ApoE promoter reporter constructs, which were co-transfected with either Rela expression vector or empty vector into neurons. The data are presented as the ratio of luciferase activity from the empty-vector- versus Rela-expression-vector-transfected neurons. (K) ChIP-PCR results of the unedited and $Rela^+$ (FLAG-tagged) neurons. The data are expressed by the results of qRT-PCR (relative to 1% of input) for the pulled-down DNA fragments using the primers around the putative binding sites of Rela in the ApoE gene. (L) Alignment of promoter-flanking region of mouse ApoE gene with the human and rat counterparts. Asterisks indicate nucleotides conserved. Putative binding sites for Rela in ApoE gene are boxed. (M and N) Immunoblotting (M) and quantitative data (N) of APP and Aβ in unedited/$Rela^+$ neurons after injury and exosome treatment. n = 6 per group. The data are presented as mean ± SD. $^{*}p < 0.05$, $^{**}p < 0.01$, and $^{***}p < 0.001$ versus the I+EXO-124 group; $^{\#\#}p < 0.01$ and $^{\#\#\#}p < 0.001$ versus the control group. Abbreviations: ChIP-F, forward primer for ChIP assay; ChIP-R, reverse primer for ChIP assay; DPI, days post-injury; I(un), unedited injured neurons; I($Rela^+$), Rela overexpressed injured neurons; Luciferase-F, forward primer for luciferase assay; Luciferase-R, reverse primer for luciferase assay; Mut, mutant type; WT, wild-type.

Table 1. mRNA Expression Level of 26 Neurodegeneration-Related Genes Potentially Targeted by Rela in Injured Neurons under the Regulation of miR-124-3p

	Fold Change	
Gene Name	Injured versus Sham	I+124 Mimics versus Injured
Agt1	0.89	1.04
Akt1	1.26	0.93
ApoE	0.23[a]	3.29[a]
Bcl2	0.87	1.01
Ccl2	0.68	1.06
Ccl20	0.36[a]	0.70
Ccnd1	1.05	0.95
Cd38	0.82	0.68
Cd40	0.87	0.71
Cxcl10	0.67	1.09
Fas	0.83	0.96
Hgf	1.52	0.66
Ifng	3.11[a]	2.75[a]
Mmp14	1.12	1.07
Mmp9	0.86	0.69
Myc	1.04	1.12
Ngb	1.02	1.03
Nos2	0.53	0.49[a]
Ppara	1.04	1.82
Ptgs2	0.95	1.30
Selp	3.21[a]	3.14[a]
Shh	1.05	0.78
Slc1a2	1.04	0.89
Tlr2	0.56	0.71
Trp53	1.16	1.25
Trp73	0.89	1.04

[a]Indicates fold change more than 2 or less than 0.5, with a $p < 0.01$.

(Figure 7C). Therefore, EXO-124 treatment could improve the spatial learning and memory abilities of rmTBI mice (Figure 7D). In the novel object recognition test, the amount of time taken to explore the new object provides an indicator for cognitive memory. We found that the index of exploring time on the novel object over the total exploring time was decreased in the rmTBI mice compared with that in the sham mice. In addition, EXO-124 treatment improved the index of exploring time on novel object, suggesting that it contributed to the recovery of cognitive function after rmTBI. Taken together, these findings suggested that EXO-124 treatment can improve the cognitive outcome of rmTBI mice.

DISCUSSION

With an increasing number of reported clinical cases, rmTBI-induced long-term neurodegenerative disorder has raised wide attention in recent years. Aβ abnormalities are one of the characteristic pathological changes of post-traumatic neurodegeneration.[8] However, unlike that in AD, the mechanism underlying the development of Aβ abnormalities in rmTBI has not been well elucidated. The present research is the first report that focused on studying the effect and mechanism of microglial exosomal miRNAs on regulating neurodegeneration after rmTBI through controlling Aβ abnormalities. Our major discoveries are as follows: (1) the miR-124-3p level in microglial exosomes from injured brain is significantly altered in the acute, sub-acute, and chronic phases after rmTBI. (2) EXO-124 treatment for cultured neurons after repetitive injury alleviates neurodegeneration by promoting neurite outgrowth, regulating the expression of neurodegenerative indicators, and inhibiting Aβ abnormalities. (3) The impact of microglial exosomal miR-124-3p is exerted by targeting Rela, an inhibitory transcription factor of ApoE that promotes the Aβ proteolytic breakdown, thereby inhibiting Aβ abnormalities. (4) The microglial exosomes that were injected into rmTBI mice can be taken up by neurons in injured brain. EXO-124 treatment contributes to alleviating neurodegeneration by targeting the Rela/ApoE signaling pathway in hippocampal neurons, thus improving the cognitive outcome of rmTBI mice.

In previous studies of neurodegenerative diseases, the microglial exosomes have been described as a double-edged sword for the development of Aβ abnormalities and neurodegeneration.[13] Take AD as an example: the microglia activation has beneficial effects in the early stage of AD, as it induces phagocytosis and Aβ clearance with the help of microglial exosomes. However, in the latter stage of AD, microglial exosomes exert detrimental effects on neurons, because they contain and spread pro-IL-1β, caspase-1, and soluble toxic Aβ peptide in brain. Our findings confirm a similar viewpoint in rmTBI from the perspective of miRNA regulation. We find that the expression level of miR-124-3p was increased in rmTBI mice brains since 1 DPI, reached the peak at 7–14 DPI, then gradually decreased to the baseline at 35 DPI, and finally dropped down to almost half below the baseline at 42 DPI. As miR-124-3p in microglial exosomes has been proved to be beneficial for alleviating Aβ abnormalities and neurodegeneration, this suggested that an increased miR-124-3p level in the acute phase (1–7 DPI) and sub-acute phase (14–28 DPI) of rmTBI contributed to the beneficial impacts of microglial exosomes, while a decreased miR-124-3p level in the chronic phase (42 DPI) of rmTBI could lead to detrimental effects. From this aspect, keeping the overexpression of miR-124-3p in microglia and their exosomes is a promising therapeutic strategy for rmTBI. Our designed EXO-124 treatment that contributes to alleviating neurodegeneration and improving the cognitive outcome after rmTBI complies with this theory.

miR-124-3p is a critical modulator of immunity and inflammation. It is the most expressed miRNA in brain neural cells and is highly expressed in the immune cells and organs.[39] miR-124-3p serves as a universal regulator of macrophage polarization, which could switch microglia from M0 or M1 to M2 subsets.[40] In many cases, miR-124-3p is induced by inflammatory signals and, in turn, functions

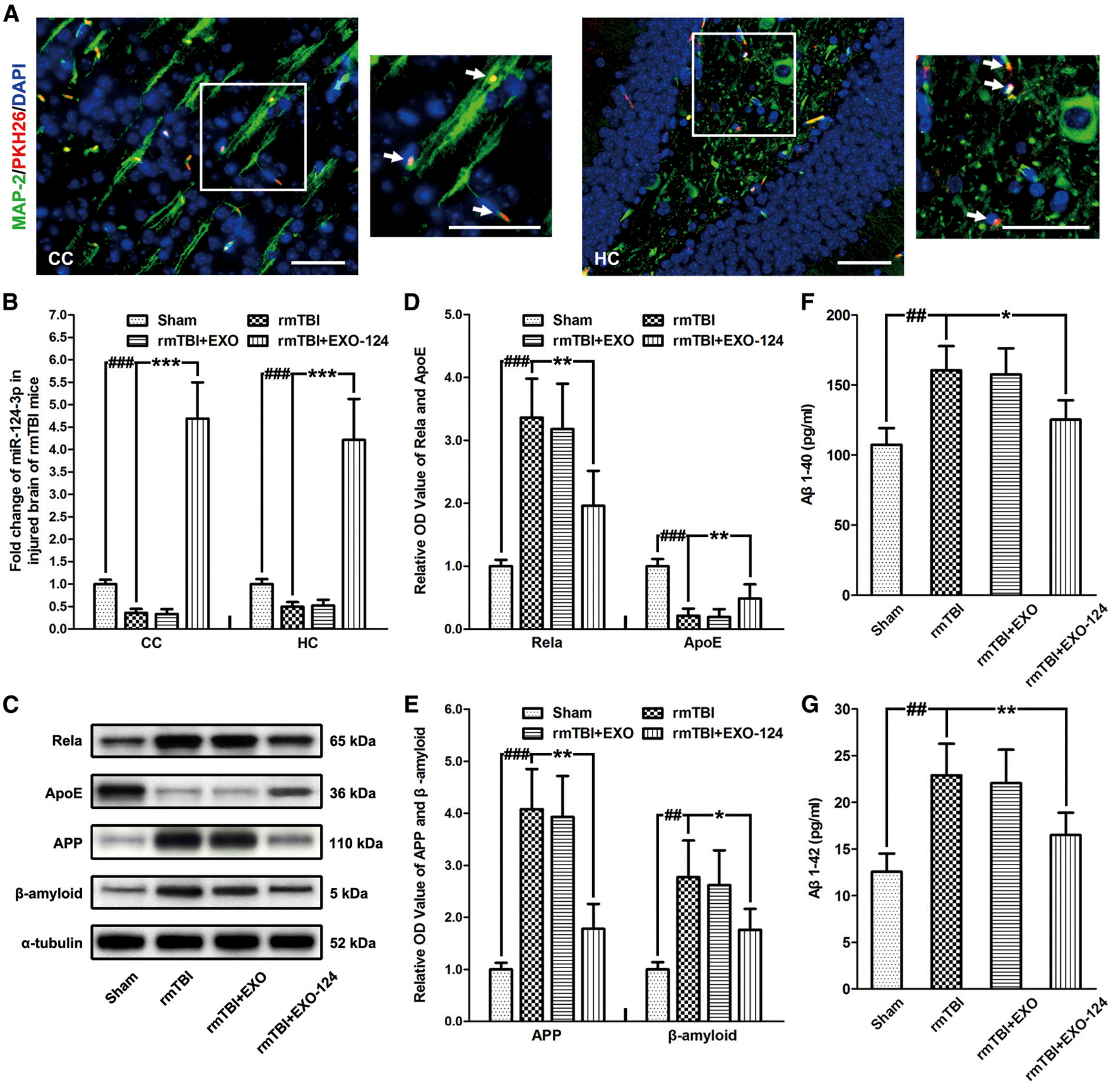

Figure 6. Microglial Exosomes with Upregulated miR-124-3p Contribute to Alleviating Neurodegeneration in rmTBI Mice

(A) Immunofluorescence staining of neurons in the cerebral cortex (CC) and the hippocampus (HC) of rmTBI mice, taking up exogenous microglial exosomes. Note that the intravenously injected exosomes are taken up by neurons (marked by co-expression of MAP-2 and PKH26) at 42 days post-injury (DPI). The immunostained areas in the white box are shown with high magnification. The double immunostained cells are indicated by white arrows. Scale bars: 50 μm. (B) The altered miR-124-3p level in the CC and the HC of rmTBI mice after exosome treatment at 42 DPI. (C) Immunoblotting of Rela, ApoE, APP, and Aβ in the HC of rmTBI mice after exosome treatment at 42 DPI. (D) Quantitative data of Rela and ApoE in the HC of rmTBI mice after exosome treatment at 42 DPI. (E) Quantitative data of APP and Aβ in the HC of rmTBI mice after exosome treatment at 42 DPI. (F and G) The expression levels of soluble (F) $A\beta_{1-40}$ and (G) $A\beta_{1-42}$ in brain extracts from HC at 42 DPI. n = 6 per group. The data are presented as mean ± SD. *p < 0.05, **p < 0.01, and ***p < 0.001 versus the rmTBI+EXO-124 group; ##p < 0.01 and ###p < 0.001 versus the sham group.

as a negative regulator to maintain homeostasis.[39] Therefore, the expression changes on miR-124-3p is considered as a biomarker for neuroinflammation in neurological diseases.[41,42] Of particular note, chronic neural inflammation, which has been confirmed to contribute to the development of post-traumatic neurodegeneration, can be detected in rmTBI patients.[43] In the present study, upregulated miR-124-3p in microglia promotes their anti-inflammatory M2 polarization *in vitro*. Besides, the Rela gene, which is responsible for encoding the p65 subunit of nuclear factor κB (NF-κB) that plays a central role in activating inflammatory response,[44] is also targeted and inhibited by microglial exosomal miR-124-3p in injured neurons. These results are consistent with our previous findings that the increased miR-124-3p in microglial exosomes following TBI inhibits neuronal inflammation.[23] Furthermore, we find that intravenously injected microglial exosomes could be taken up by astrocytes and microglia in addition to neurons in the injured brain. A recent report indicated that mesenchymal-stem-cell (MSC)-derived exosomes have therapeutic effects on ischemic stroke due to their regulative

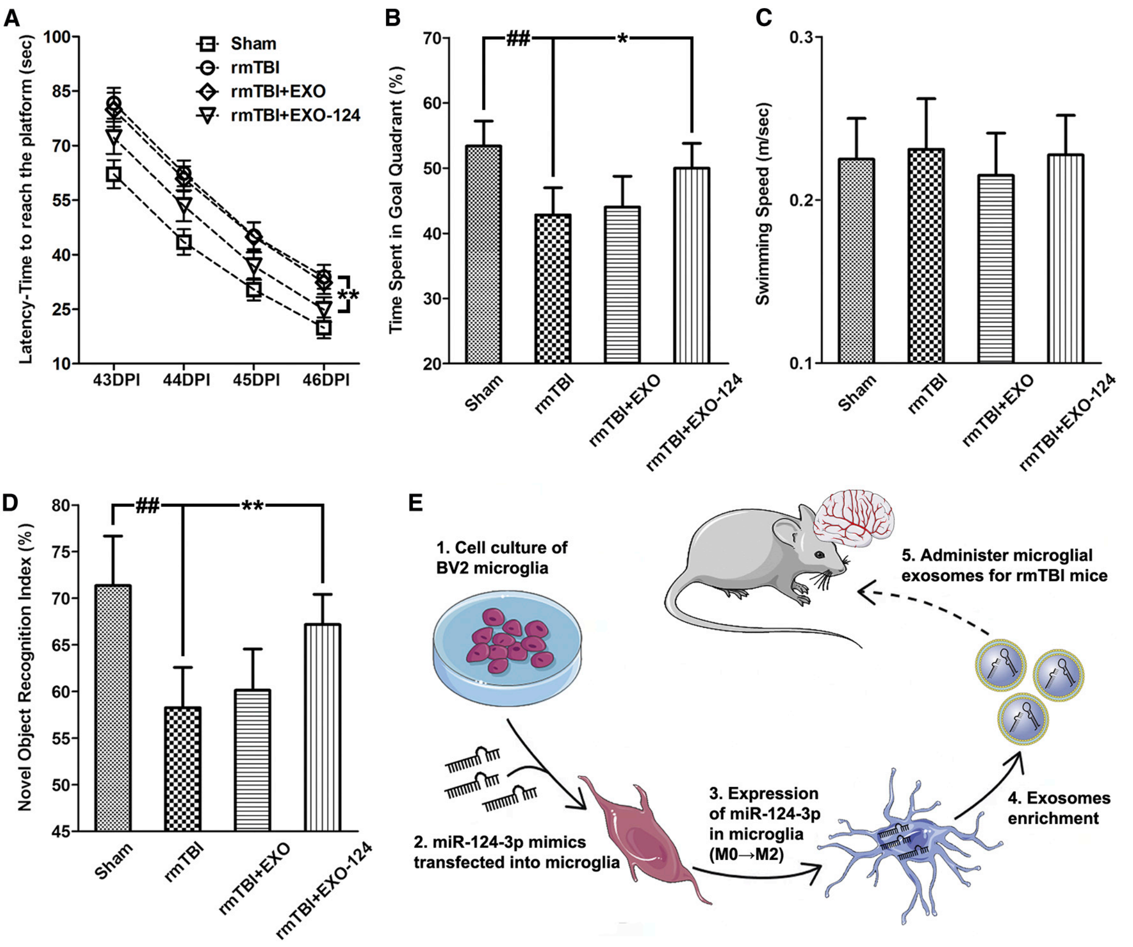

Figure 7. Microglial Exosomes with Upregulated miR-124-3p Improve the Cognitive Outcome of rmTBI Mice

(A) The escape latency detected in the spatial acquisition trials (43–46 days post-injury [DPI]) of the Morris water maze (MWM) test. (B) The time spent in the goal quadrant detected in the probe trials (47 DPI) of the MWM test. (C) The swimming speed of rmTBI mice in the MWM test. (D) The novel object recognition index of rmTBI mice detected at 42 DPI. (E) The schematic illustration of the *in vivo* experiment. n = 10 per group. The data are presented as mean ± SEM in (A) and mean ± SD in (B)–(D). *p < 0.05 and **p < 0.01 versus the I+EXO-124 group; $^{##}$p < 0.01 versus the control group.

functions on the activation of astrocytes and microglia. It suggests that exogenous MSC-derived exosomes might be taken up by these cells, thus modulating the immunoinflammatory response in brain.[45] From this, we infer that EXO-124 could also produce anti-inflammatory effects after rmTBI via transferring into astrocytes and microglia. The related mechanism will be studied further in our follow-up study. Nevertheless, these findings further confirm our conclusion that miR-124-3p in microglia and their exosomes could exert a protective effect in injured brain after rmTBI.

The ApoE protein is expressed as three different isoforms resulting from the different polymorphisms in the ApoE gene—designated as ApoE2, ApoE3, and ApoE4—which are differed by single amino-acid substitutions at residues 112 and 158.[46] Their protective effects on alleviating neuroinflammation and neurodegeneration are different from each other, with a potency rank of ApoE2 > ApoE3 > ApoE4.[47] Despite the gene polymorphism, the ApoE proteins suppress glial activation and inflammatory cytokine release *in vitro*.[48] These findings could also be extended to TBI, in which the absence of endogenous ApoE exacerbates post-injury neuroinflammation, cerebral edema, and neurological deficiency.[49] Besides, ApoE enhances the proteolytic breakdown of Aβ in AD and rmTBI.[37,50] As to the present study, the expression of ApoE in injured neurons is promoted by microglial exosomal miR-124-3p via targeting Rela, which functions as an inhibitory transcription factor. In rmTBI mice, EXO-124 treatment also promotes ApoE expression in hippocampal neurons. Consequently, ApoE is an extraordinarily significant gene for regulating Aβ abnormalities in neurodegenerative diseases.

In addition to the mechanism we clarified in this research, microglial exosomes are involved in the pathological regulation of neurodegenerative diseases in several ways. First, microglial exosomes could be released in response to WNT3A, which is a signaling factor that

has been implicated in several neurodegenerative diseases, including AD.[51] Besides, microglial exosomes are also reported to aggravate AD by contributing to the propagation of tau protein.[52] In addition, the release of exosomes from microglia is influenced by neurotransmitters. Stimulation of the 5-hydroxytryptamine receptor on microglia could lead to the release of exosomes containing an insulin-degrading enzyme that targets Aβ peptide.[53] It explains the phenomenon that an increased level of 5-hydroxytryptamine is associated with a decreased level of Aβ in brain tissue of AD.[54] Therefore, future studies on the roles of microglial exosomes in rmTBI could be designed to refer to AD research.

This study is still limited in learning the pharmacokinetics and pharmacodynamics parameters of EXO-124 treatment for rmTBI mice. To address the questions, we are performing translational experiments in rmTBI mice using EXO-124 treatment with different doses and administration time. In addition, a series of studies on MSC-derived exosomes for TBI treatment have been reported recently, indicating that they could effectively improve the neurological outcome by promoting endogenous angiogenesis and neurogenesis while attenuating neural inflammation.[55–57] Therefore, the therapeutic effect of MSC-derived exosomes with upregulated miR-124-3p for rmTBI will be evaluated and compared to that of microglial exosomes in future studies.

In conclusion, our findings suggest that microglial exosomal miR-124-3p contributes to alleviating neurodegeneration and improving the cognitive outcome after rmTBI via transferring into neurons and targeting the Rela/ApoE signaling pathway. Consequently, EXO-124 treatment is a promising therapeutic strategy for rmTBI with clinical application prospects.

MATERIALS AND METHODS

Adult male C57BL/6 mice (aged 12 weeks, weighing 20–25 g) were purchased from the Chinese Academy of Military Science (Beijing, China). All experimental procedures were performed in accordance with the NIH Guide for the Care and Use of Laboratory Animals under a protocol approved by the Tianjin Medical University Animal Care and Use Committee. The mice were acclimated and housed for 1 week before experiments.

CCI-Induced rmTBI Mouse Model

The rmTBI mouse model was conducted using our developed method, which could lead to chronic neurodegeneration and cognitive dysfunction within 5–6 weeks post-injury.[22] Briefly, the mice were subjected to closed-head injury after anesthesia with 4.6% isoflurane. A molded acrylic cast was designed to fix the mice and provide their heads with a 3.0-mm space below for acceleration and deceleration beneath the point of impact. The mouse was secured in the prone position on the acrylic cast with surgical tape across the shoulders, which was installed on the stereotaxic device. After shaving the head, a self-designed standard manufacturing concave metal disc was adhered to the skull immediately caudal to bregma as a helmet, in order to transmit the hitting power to the whole brain. Specifically, scalp incision was not performed to better stimulate the injured procedure of rmTBI patients. The impounder tip of the injury device (electronic controlled cortical impact [CCI] device, model 6.3, American Instruments, Richmond, VA, USA) was then extended to its full impact distance, positioned on the center of the disc surface, and reset to induce an impact. It was discharged at 5.0 m/s with a head displacement of 2.5 mm. After the impact, the mice were placed in a well-ventilated cage at 37°C until they regained consciousness. Repetitive injury was induced for a total of 4 times, with a 48-h interval. Sham-operated mice underwent the same procedures except for the impact. To determine the degree of brain injury, H&E staining was performed on brain sections at 1 DPI.

Isolation of Microglial Exosomes from Injured Brain

The animals were sacrificed at 1, 3, 7, 14, 21, 28, 35, and 42 DPI by transcardiac perfusion with PBS. To acquire the desired samples from injured brain, we detached the bilateral cerebral cortex and hippocampus, mixed them together, and incubated them with 3 mL Hibernate-A cell culture medium (Thermo Fisher Scientific, Waltham, MA, USA). The samples were digested by 20 U/mL papain (Solarbio, Beijing, China) for 15 min at 37°C, and repeated blowing was performed using a dropper to remove the tissue fragment. They were then centrifuged at 2,000 × *g* for 10 min at 4°C and then spun again at 10,000 × *g* for 30 min at 4°C to further remove the cell debris. The supernatants were collected and filtered through a 0.22-μm filter gauze (Millipore Sigma, Billerica, MA, USA) to sieve out dead cells and large particles. After that, Total Exosome Isolation Reagent (Thermo Fisher Scientific) was added to the supernatants at the proportion of 1 mL supernatant to 500 μL reagent, and the samples were incubated overnight at 4°C.

On the next day, the exosomes were harvested by ultracentrifugation. First, the sample was ultracentrifuged at 100,000 × *g* for 70 min at 4°C. The supernatants were then removed, and the exosome pellets were collected and re-suspended in 0.35 μL calcium- and magnesium-free Dulbecco's PBS (Thermo Fisher Scientific). For isolation of microglial exosomes, the samples were incubated with 1.5 μg rat anti-mouse CD11b bitinylated antibody (Thermo Fisher Scientific) in 50 μL 3% BSA for 60 min at room temperature. After that, they were incubated with 10 μL Pierce Streptavidin Plus UltraLink Resin (Thermo Fisher Scientific) in 40 μL 3% BSA for 30 min at room temperature. Finally, the samples were centrifuged at 800 × *g* for 10 min at 4°C. After removal of the supernatants, the microglial exosomes could be stored at 4°C temporarily for subsequent experiments.[58]

Exosome Identification

For exosome identification, TEM (Hitachi HT7700, Tokyo, Japan) scanning was performed to observe the morphology of precipitated particles as we previously reported.[23] Briefly, the isolated precipitation from injured brain was diluted into distilled water (1 μg/μL) and mixed with same amount of 4% paraformaldehyde (PFA). 20 μL of the sample was added onto a glow-discharged, carbon-coated formvar film that attached to a metal specimen grid. The grid was incubated with 50 μL 1% glutaraldehyde for 5 min at room

temperature and washed with 100 μL distilled water for 8 times (2 min each time). After drying for 30 min with filter papers, an equal volume of 10% uranyl acetate was added to the grid for 5 min at room temperature, followed by 50 μL methyl cellulose-uranyl acetate (5 μL 4% uranyl acetate and 45 μL 2% methyl cellulose) for 10 min at 4°C. Then, the excess solution was blotted off, and the sample was examined using the TEM. In addition, size distribution of the precipitated particles was measured and analyzed using the Nanosight NS300 NTA System (Malvern Panalytical, Malvern, UK) according to the manufacturer's instructions. Biomarkers for exosomes, including CD9, CD63 and CD81, were also detected by western blot for further identification. To extract proteins in microglial exosomes, the exosomes were re-suspended in 100 μL 0.05 M glycine-HCl (pH 3.0) and centrifuged at 4,000 × *g* for 10 min at 4°C. The supernatants were then transferred to pre-chilled tubes that contained 25 μL 10% BSA and 10 μL 1 M Tris-HCl (pH 8.0) and were mixed gently with the addition of 365 μL M-PER Mammalian Protein Extraction Reagent (Thermo Fisher Scientific) containing protease and phosphatase inhibitors for 10 min.[59] The samples could be stored at −80°C for following protein detection.

miRNA Microarray Analysis

Microglial exosomes acquired from the injured brain were sent for miRNA microarray analysis (OE Biotechnology, Shanghai, China). Total RNA was quantified by the NanoDrop ND-2000 (Thermo Fisher Scientific), and the RNA integrity was assessed using the Agilent Bioanalyzer 2100 (Agilent Technologies, Santa Clara, CA, USA). The sample labeling, microarray hybridization, and washing were performed in accordance with the manufacturer's standard protocols. Briefly, total RNA was dephosphorylated, denaturated, and labeled with cyanine-3-CTP. After purification, the labeled RNAs were hybridized onto the microarray that contains 1,902 probes for mature miRNA. Then, the arrays were scanned with the Agilent Scanner G2505C (Agilent Technologies).

We selected 3, 14, and 42 DPI to represent, respectively, the time points of acute phase, sub-acute phase, and chronic phase after rmTBI. The miRNAs with level change of more than 2-fold (up- or downregulated compared with the Sham group) at the 3 time points ($p < 0.01$) were screened out, and qRT-PCR was performed to validate the expression changes of miR-124-3p in injured brain after rmTBI.

Cell Culture of BV2 Microglia and miR-124-3p Mimic Transfection

The BV2 mouse microglial cell line was obtained from Nankai University (Tianjin, China). The cells were seeded into 6-well plates at the density of $5 \times 10^5/cm^2$ and cultured in DMEM/F12 culture medium containing 10% fetal bovine serum (FBS), 100 U/mL penicillin, and 100 mg/mL streptomycin (GIBCO Laboratory, Grand Island, NY, USA) at 37°C. The purity of cultured microglia was determined by immunofluorescence staining of Iba-1 (a biomarker of microglia).

To investigate the function of miR-124-3p in microglial exosomes, miR-124-3p mimics (sequences: 5′-UAAGGCACGCGGUGAAUG CCCA-3′, GenePharma, Shanghai, China) were transfected into microglia as we previously described.[60,61] Briefly, miR-124-3p mimics were first diluted to 20 μM. 5 μL miR-21-3p mimic was then incubated with an equal volume of Lipofectamine 3000 (Invitrogen, Carlsbad, CA, USA) in 500 μL serum-free DMEM/F12 medium for 20 min at room temperature. This transfect solution was added into the culture plates for 6 h of transfection, followed by replacing with the complete DMEM/F12 medium. The miR-124-3p upregulated exosomes were harvested from the culture medium 48 h later. To evaluate the transfection efficacy, qRT-PCR was performed to detect the level change of miR-124-3p in microglia and their exosomes.

Enrichment of Exosomes from Cultured Microglia

The culture medium of microglia was first collected into the 50-mL polypropylene tube and centrifuged at 300 × *g* for 10 min at 4°C to remove free cells. The supernatants were then transferred into a fresh centrifuge tube, spun at 2,000 × *g* for 10 min at 4°C to remove cell debris, and then spun again at 10,000 × *g* for 30 min at 4°C to further remove additional cell particles. After that, the supernatants were filtered through a 0.22-μm filter gauze to sieve out dead cells and large particles. Total Exosome Isolation Reagent was added to the supernatants at the proportion of 1 mL supernatant to 500 μL reagent. The samples were mixed by vortexing and were incubated overnight at 4°C. On the next day, ultracentrifugation was performed at 100,000 × *g* for 70 min at 4°C. After removal of the supernatants, the exosomes were stored at 4°C temporarily for further experiments.

Flow Cytometry

To study the impact of miR-124-3p on microglial polarization, flow cytometry was performed to measure the ratio between M2 and M1 microglia in cultured cells after 24 h of miR-124-3p mimic transfection. Briefly, single-cell suspensions of microglia were co-stained for CD206 (an M2 microglia biomarker) conjugated with allophycocyanin (APC) (0.5 μg per test; 141-708; Thermo Fisher Scientific) and CD86 (an M1 microglia biomarker) conjugated with fluorescein isothiocyanate (FITC) (1.0 μg per test; 105110; Thermo Fisher Scientific) for 45 min at room temperature following the manufacturer's instructions. The samples were detected with the FACSAria III flow cytometer (BD Biosciences, San Jose, CA, USA). Subsequent data analysis was performed using FlowJo software v.7.6.1 (https://flowjo.com/).

Cell Culture of HT22 Hippocampal Neurons and Establishment of Repetitive Scratch Injury Model

The HT22 mouse hippocampal neuronal cell line was obtained from the Tianjin Cancer Institute (Tianjin, China). The cells were seeded into 6-well plates pre-coated with poly-D-lysine (MilliporeSigma) at the density of 5×10^5 per well and cultured in the neurobasal medium containing 10% FBS, 2% B27, and 1% glutamine (GIBCO Laboratory) at 37°C. The purity of cultured neurons was determined by immunofluorescence staining of MAP-2 (a biomarker of neuron).

To study the impact of microglial exosomes on neurons after rmTBI *in vitro*, the repetitive scratch injury model was designed in which two scratch injuries were conducted with a 12-h interval. The first injury

was performed by scratching across the cell surface vertically with a 4-mm space between each line using a 10 μL pipette tip. The second injury was performed using the same method but scratching horizontally. After each scratch injury, the detached cells were removed by washing with the neurobasal medium (GIBCO Laboratories).

The impact of repetitive scratch injury on the cell viability of neurons was evaluated by CCK8 assay following the manufacturer's instructions (Dojindo Laboratories, Tokyo, Japan). The measurements were performed every 3 h from the first injury to 24 h after the second injury. The result of optical density (OD) values, which represent the number of viable cells, was detected with the Varioskan Flash Microplate Reader (Thermo Fisher Scientific) at the wavelength of 450 nm.

Microglial Exosome Treatment for Cultured Neurons

The cultured neurons were randomly assigned into four groups: uninjured neurons (control), injured neurons (injury), injured neurons treated with unedited microglial exosome (I+EXO), and injured neurons treated with EXO-124 (I+EXO-124). For exosome treatment, the culture medium of neurons was replaced with the serum-free neurobasal medium right after the second scratch injury. Media containing 3×10^8 microglial exosomes were then added to the culture plate.[23,62] To evaluate the transfer efficacy of miR-124-3p via exosomes, qRT-PCR was performed to detect the expression level of miR-124-3p in neurons after 24 h.

Neurodegeneration Evaluation for Cultured Neurons after Injury

We evaluated neurodegeneration for repetitive injured neurons after exosome treatment from three aspects: neurite outgrowth, expression of degenerative indicators, and Aβ abnormalities.

For neurite outgrowth detection, immunofluorescence staining of neuronal-specific beta III-tubulin (also called Tuj1) was performed at 24 h after injury and exosome treatment. Beta III-tubulin-positive cells were digitized under a 20× objective using a 3-charge-coupled device (CCD) color video camera (Sony DXC-970MD, Tokyo, Japan) with an immunofluorescence microscope (Olympus IX81, Tokyo, Japan). The numbers and total length of neurite branches were quantified using the NIS-Elements BR Analysis System (Nikon, Tokyo, Japan). At least 60 beta III-tubulin-positive cells, distributed in 9 random fields per well, and triple wells per group were measured.

To further investigate whether miR-124-3p could promote neurite outgrowth, the injured neurons were transfected with miR-124-3p mimics for 12 h and then were rinsed with PBS for 3 times and transfected with miR-124-3p inhibitor (sequences: 5′-GGCAUUCACCG CGUGCCUUA-3′, GenePharma) for another 12 h (for a schematic representation, see Figure 3D). During the injury and transfecting procedure, the changes on neuronal branches were observed continuously using HPICM as previously described.[23] Specifically, the neurons for HPICM scanning were scratched crosswise, and the scanning time was controlled within 24 h; otherwise, the cell viability will be influenced by the scanning procedure. The HPICM was composed with a sample scan head SH01 and an ICnano scanner controller (Ionscope, Melbourn, Cambridgeshire, UK). The SH01 with a nanopipette was placed on the inverted TiU microscope (Nikon). The ICnano controller managed two 100-μm PIHera piezo nanopositioners (P-621.2C, Physik Instrumente, Karlsruhe, Germany) to control cell movement in the horizontal X-Y direction. In addition, a 25-μm LISA piezo nanopositioner (P-753.21C, Physik Instrumente) was used to control vertical positioning and hopping of the probe. The Axon Multi Clamp700B amplifier (Molecular Devices, Sunnyvale, CA, USA) provided +200 mV of DC voltage between the nanopipette electrode and the bath electrode and monitored the ion current between the nanopipette tip and cell surface. When the nanopipette tip approached the neuron membrane, a 0.4% reduction of reference DC currents was set to keep the pipette away from the cell surface. The primary topography data were processed and analyzed using SICM Image Viewer software (Ionscope).

For degenerative indicator measurement, the expression levels of BDNF, neurogranin, and VILIP-1 in neurons were detected by western blot at 24 h after injury and exosome treatment.

Aβ abnormalities are one of the characteristic pathological changes of post-traumatic neurodegeneration.[8] Thus, we detected the expression levels of APP and Aβ protein in neurons by western blot and measured the soluble $A\beta_{1-40}$ and $A\beta_{1-42}$ levels in the culture medium by ELISA (KMB3481 and KMB3441; Thermo Fisher Scientific) at 24 h after injury and exosome treatment.

Target Prediction of miR-124-3p and Bioinformatics Analysis

Target predication for miR-124-3p was carried out using TargetScan (http://targetscan.org/), miRanda (http://www.microrna.org/microrna/home.do) and miRDB (http://mirdb.org/miRDB/). The intersection of the predicted genes from the three databases was thus obtained, and those that related to the process of neurodegeneration or amyloid deposition were further picked out using the OMIM database (https://www.omim.org/). The genetic interaction network of miR-124-3p with these genes was built by Biovista Vizit (https://biovista.com/vizit/#goviral). The KEGG analyses for miR-124-3p targeted genes were performed using Cytoscape software v.3.6.1 (https://cytoscape.org/).

Target genes of Rela (functions as a transcription factor) were predicted using the ChIP-Seq data from OmicsNet (https://www.omicsnet.ca/OmicsNet/). The predicted genes that related to the process of neurodegeneration or amyloid deposition were picked out using Ingenuity Pathway Analysis. Their mRNA expression levels were then detected in uninjured, injured, and miR-124-3p mimic-transfected injured neurons by qPCR microarray (Sheweisi Tech, Tianjin, China).

Luciferase Reporter Assay

To verify whether miR-124-3p directly targeted Rela mRNA in neurons, the luciferase reporter constructs were made by ligating the Rela 3′ UTR fragments containing the predicted miR-124-3p binding sites (TargetScan; http://www.targetscan.org/vert_71/) into a

luciferase reporter vector, pGL-3.0 (Promega, Madison, WI, USA), as we previously reported.[23,63] Specifically, the Rela 3′ UTR fragments were amplified by PCR (primer sequences: forward, 5′-TCTAGACGGTCCTTCAGTTTTTGTGC-3′; reverse, 3′-TCTAGAAACAGCACACTAAACTACCACG-5′) and were inserted into the pGL-3.0 vector using the XbaI sites immediately downstream of the luciferase coding sequence to generate the WT luciferase reporter. In addition, the Mut luciferase reporter was generated from the WT by deleting the binding sites for miR-124-3p using the KOD-Plus-Mutagenesis Kit (Toyobo, Osaka, Japan). For the following reporter assay, the WT or Mut Rela-3′ UTR was co-transfected with 200 pmol miR-124-3p mimics or scrambled oligonucleotides (GenePharma) into neurons using Lipofectamine 3000. After 48 h of incubation, the cells were harvested, and the luciferase activity was measured using a dual-luciferase reporter system (Promega, Madison, WI, USA) according to the manufacturer's instructions.

To determine whether Rela targets ApoE promoter in neurons, we got the promoter sequences from the ENCODES project analyzed by the UCSC Genome Browser (http://genome.ucsc.edu/cgi-bin/hgTables). The sequences of the promoter-flanking region of ApoE from mouse, human, and rat were then aligned by Mulan (https://mulan.dcode.org/) and Clustal Omega (https://www.ebi.ac.uk/Tools/msa/clustalo/). The ApoE promoter reporter constructs were made by ligating the DNA fragments containing the predicted Rela binding sites in the ApoE gene (PROMO, http://alggen.lsi.upc.es/cgi-bin/promo_v3/promo/promoinit.cgi?dirDB=TF_8.3; GTRD, http://gtrd.biouml.org/), which were amplified by PCR (primer sequences: forward, 5′-GGTACCCAAACCCATCGGGAGCCACC-3′; reverse, 3′-GAGCTCCCGAGTTTTTTCGCCATAGG-5′) into the pGL-3.0 promoter vector. The corresponding Mut reporter that deleted Rela binding sites was generated using the KOD-Plus-Mutagenesis Kit. For the following reporter assay, the WT or Mut ApoE reporter constructs were co-transfected with a Rela expression vector or an empty vector (Youbio, Changsha, China) into neurons using Lipofectamine 3000. After 6 h of transfection, the cell medium was changed, and the luciferase activity was detected at 48 h after transfection using a dual-luciferase reporter system.

ChIP-PCR Assay

To verify whether Rela could directly target the ApoE gene as a transcription factor, the ChIP-PCR assay was performed in two ways using the EZ-Magna ChIP G Kit (MilliporeSigma).

In the first experiment, the Rela expression vector (Youbio) was first modified to be FLAG-tagged and transfected into cultured neurons. After establishing a stable cell line of FLAG-tagged Rela overexpressed neurons, the cells were incubated with 1% PFA to cross-link proteins with DNAs. Sonication was then performed to shear the genomic DNAs to a length under 500 bp. For the immunoprecipitation step, approximately 5×10^5 neurons were incubated with 2-μg anti-FLAG tag antibody. A same amount of rabbit immunoglobulin G (IgG) was used as the negative control.

In the second experiment, unedited neurons were directly incubated with the primary antibody of Rela (1:1,000; 3670; CST; Danvers, MA, USA) after the procedures of cross-link and sonication. The protein G conjugated agarose beads were then used to mutually enrich the protein and DNA complexes. Rabbit IgG was used as the negative control.

The immunoprecipitated chromatins acquired from the two experiments were incubated in 5 M NaCl at 65°C overnight to reverse the cross-links. On the next day, the pulled-down DNA fragments were digested with proteinase K and were recovered by phenol/chloroform extraction and ethanol precipitation. The precipitated DNAs were then amplified (primer sequences: forward, 5′-GGGTTACCTCCAGGAAGGAG-3′; reverse, 3′-CATGAGGAGCCACAGTTTGA-5′), and enrichment was measured by qRT-PCR.

Analysis for the Function of the Rela/ApoE Signaling Pathway in Neurodegeneration under miR-124-3p Regulation

To validate the regulative effect of miR-124-3p on neurodegeneration through the Rela/ApoE signaling pathway, we detected the expression levels of ApoE, APP, and Aβ in three groups of neurons at 24 h after injury and exosome treatment by western blot: (1) unedited neurons after injury, (2) unedited neurons after injury and EXO-124 treatment, and (3) $Rela^+$ neurons after injury and EXO-124 treatment.

Microglial Exosome Treatment for rmTBI Mice

In our *in vivo* study (for a schematic illustration, see Figure 7E), the mice were randomly assigned into four groups: sham mice, rmTBI mice, rmTBI mice treated with unedited microglial exosome (rmTBI+EXO), and rmTBI mice treated with EXO-124 (rmTBI+EXO-124). Before treatment, the exosomes were labeled using PKH26 Fluorescent Cell Linker Kits (MilliporeSigma) according to the manufacturer's protocol, and their numbers were counted using a qNano particle analyzer (Izon, Cambridge, MA, USA). The PKH26-labeled microglial exosomes were then intravenously injected into rmTBI mice via tail vein (3×10^{10} in 200 μL PBS per mouse) at 35 DPI.[38] To examine the uptake of exosomes by neurons, astrocytes, and microglia in injured brain, an immunofluorescence staining of MAP-2, GFAP, and Iba-1 was performed separately on the brain sections of rmTBI+EXO mice at 42 DPI. In addition, qRT-PCR was performed to detect the miR-124-3p level in injured brain at 42 DPI in order to further evaluate the transfer efficacy of exogenous miR-124-3p via exosomes.

Neurodegeneration Evaluation and Rela/ApoE Signaling Pathway Detection for rmTBI Mice

To further confirm the regulative effect of microglial miR-124-3p on neurodegeneration through the Rela/ApoE signaling pathway *in vivo*, the expression levels of Rela, ApoE, APP, and Aβ in the hippocampus of rmTBI mice after exosome treatment were detected at 42 DPI by western blot. In addition, soluble $A\beta_{1-40}$ and $A\beta_{1-42}$ levels in the brain extracts of hippocampus were measured at 42 DPI by ELISA (KMB3481 and KMB3441; Thermo Fisher Scientific).

Tissue Preparation for *In Vivo* Sample Measurement

For immunofluorescence staining, the mice were sacrificed by transcardiac perfusion with PBS followed by 2% PFA, 10% sodium periodate, and 2% 70 mM L-lysine. The brains were then dissected and post-fixed in the same solution for 1 h and incubated in 30% sucrose overnight. After fixation, they were embedded in the optimum cutting temperature medium (Sakura, Torrance, CA, USA). Coronal sectioning was performed on a cryostat, and the sections were stored at −20°C.

For qRT-PCR, western blot, and ELISA, the mice were sacrificed by transcardiac perfusion with only PBS. The bilateral cerebral cortex was first acquired from the injured brain. The bilateral hippocampus was then detached and mixed together. The brain tissue was stored in liquid nitrogen for the following experiments.

Immunofluorescence Staining

The cell or brain sections were first fixed in 4% PFA for 15 min at room temperature, followed by treating with 3% BSA for 30 min at 37°C to block nonspecific staining. They were then incubated overnight at 4°C with primary antibodies: Iba-1 (1:200; ab178847; Abcam, Cambridge, Cambridgeshire, UK), MAP-2 (1:200; ab32454; Abcam), beta III-tubulin (1:200; ab78078; Abcam), and GFAP (1:100; ZA-0117; ZSJB-BIO, Beijing, China). On the next day, the sections were rinsed by PBS and incubated with the secondary antibody for 1 h at room temperature. The nuclei were counterstained with DAPI.

Western Blot

SDS-PAGE and immunoblotting were performed as we previously described.[64] Briefly, 8% SDS-acrylamide gel was used for detecting APP (1:1,000; ab12269; Abcam). 10% SDS-acrylamide gel was used for detecting BDNF (1:1,000; ab223354; Abcam), Rela (1:1,000; 8242; Cell Signaling Technology), and ApoE (1:1,000; ab183597; Abcam). 12% SDS-acrylamide gel was used for detecting CD9 (1:2,000; ab92726; Abcam), CD63 (1:1,000; ab193349; Abcam), CD81 (1:1,000; 10037; CST), neurogranin (1:1,000; ab217672; Abcam), VILIP-1 (1:1,000; ab187631; Abcam), and Aβ (1:1,000; 15126; CST). GAPDH (1:2,000; 2118; CST) and α-tubulin (1:1,000; 2144; CST) were used as the internal control. For densitometry, the ChemiDoc XRS+ Imaging System (Bio-Rad, Hercules, CA, USA) was used. Measurement of mean pixel density of each band was conducted using Quantity One software (Bio-Rad).

Real-Time qRT-PCR

Total RNA was isolated from microglial exosomes or cultured microglia using the Total Exosome RNA and Protein Isolation Kit (Thermo Fisher Scientific) or TRIzol Reagent (Thermo Fisher Scientific) following the manufacturer's instructions. The RNA concentration was quantified by the NanoDrop ND-2000 (Thermo Fisher Scientific). Reverse transcription and qRT-PCR were performed using the Hairpin-it miR-124-3p RT-PCR Quantitation Kit (GenePharma, Shanghai, China) with corresponding primers (primer sequences: forward, 5′-TCTTTAAGGCACGCGGTG-3′; reverse, 3′-TATGGTT TTGACGACTGTGTGAT-5′) as we previously reported.[65] For microglial exosomal miR-124-3p detection, the synthetic miRNA *Caenorhabditis elegans* miR-39 (cel-miR-39; 5 fmol/μL; sequences: 5′-UCACCGGGUGUAAAUCAGCUUG-3′; GenePharma) was added to the isolated RNAs and was used as an exogenous control. For neuronal miR-124-3p detection, U6 was used as an internal control (primer sequences: forward, 5′-CTCGCTTCGGCAGCACA-3′; reverse, 3′-AACGCTTCACGAATTTGCGT-5′). The cycle threshold (Ct value) was detected by the CFX Connect Real-Time PCR Detection System (Bio-Rad). The data were analyzed using the $2^{-\Delta\Delta Ct}$ formula.

Cognitive Outcome Evaluation

All neurofunctional tests were performed in the behavioral testing room. A video-tracking system (Ethovision 3.0; Noldus Information Technology, Wageningen, the Netherlands) was utilized to record the whole procedure of animal activities in training and experimental sessions.

The MWM test was performed on rmTBI mice at 43–47 DPI. For the spatial acquisition trials, the mice were placed in a pool (105-cm diameter) filled with room-temperature water and were allowed up to 90 s to locate a submerged platform. The mice performed four trials a day with a 30-min interval for 4 consecutive days (43–46 DPI). They were introduced in varying quadrants (northwest, northeast, southwest, and southeast) of the pool for each trial, but the location of the platform was fixed. The latency—time to reach the platform—was recorded. For the probe trials that were conducted at 47 DPI (24 h after the last spatial acquisition trials), the platform was removed, and the mice were allowed to swim freely for 60 s. The percentage of the time spent in the goal quadrant was calculated.

The novel object recognition test was performed on rmTBI mice at 42 DPI as previously reported.[66,67] Briefly, the mice were allowed to freely explore a 40-cm × 40-cm × 50-cm open-field box (Clever Sys, Reston, VA, USA) for 10 min before the experiment. In the familiarization session, the mice were allowed to freely explore two similar objects. A stopwatch with two channels was used to record the time spent exploring each object until 20 s of total exploration time or 10 min of the session time was reached. In the following test session conducted 6 h later, one of the two objects was replaced by a novel object, and the mice were allowed to explore them for 10 min. The amount of time that the mice spent on exploring each object was recorded, and the index of exploring time on the novel object over the total exploring time was calculated.

Statistical Analysis

All data were based on at least three independent experiments. Except for the spatial acquisition trials of the MWM test, all data were expressed as mean ± SD and analyzed using one-way ANOVA followed by least significant difference (LSD) post hoc analysis or Student's t test. The data of spatial acquisition trials were expressed as mean ± SEM, which were analyzed using two-way ANOVA followed by LSD post hoc analysis. A p value of less than 0.05 was considered significant.

SUPPLEMENTAL INFORMATION

Supplemental Information can be found online at https://doi.org/10.1016/j.ymthe.2019.11.017.

AUTHOR CONTRIBUTIONS

J.Z., P.L., R.J., and X.G. designed the study. J.Z. and P.L. were responsible for experimental guidance. X.G. and W.L. developed the methodology. X.G., M.G., T.H., W.L., S.H., and Z.Y. carried out the experiments. Y.L. performed the HPICM scanning. F.C. and L.Z. performed the flow cytometry. C.K. provided technical support. X.G., M.G., and T.H. prepared the figures. X.G. performed data analysis and wrote the manuscript. J.Z. and P.L. reviewed the manuscript.

CONFLICTS OF INTEREST

The authors declare no competing interests.

ACKNOWLEDGMENTS

This research was supported by grants from the National Natural Science Foundation of China (grants 81501055, 81772060, 81720108015, and 81671221) and the Tianjin Municipal Science and Technology Commission Natural Fund Project (grant 18JCQNJC81100). The authors appreciate Changzheng Du from Peking University Cancer Hospital and Shu Zhang, Lei Han, Yunfei Wang, Li Liu, Weiyun Cui, and Lei Zhou from the Tianjin Neurological Institute for their technical support.

REFERENCES

1. Rubiano, A.M., Carney, N., Chesnut, R., and Puyana, J.C. (2015). Global neurotrauma research challenges and opportunities. Nature *527*, S193–S197.

2. Maas, A.I.R., Menon, D.K., Adelson, P.D., Andelic, N., Bell, M.J., Belli, A., Bragge, P., Brazinova, A., Büki, A., Chesnut, R.M., et al. (2017). Traumatic brain injury: integrated approaches to improve prevention, clinical care, and research. Lancet Neurol. *16*, 987–1048.

3. Feigin, V.L., Theadom, A., Barker-Collo, S., Starkey, N.J., McPherson, K., Kahan, M., Dowell, A., Brown, P., Parag, V., Kydd, R., et al. (2013). Incidence of traumatic brain injury in New Zealand: a population-based study. Lancet Neurol. *12*, 53–64.

4. McMahon, P., Hricik, A., Yue, J.K., Puccio, A.M., Inoue, T., Lingsma, H.F., Beers, S.R., Gordon, W.A., Valadka, A.B., Manley, G.T., et al. (2014). Symptomatology and functional outcome in mild traumatic brain injury: results from the prospective TRACK-TBI study. J. Neurotrauma *31*, 26–33.

5. Gardner, R.C., and Yaffe, K. (2015). Epidemiology of mild traumatic brain injury and neurodegenerative disease. Mol. Cell. Neurosci. *66* (Pt B), 75–80.

6. Levin, H.S., and Diaz-Arrastia, R.R. (2015). Diagnosis, prognosis, and clinical management of mild traumatic brain injury. Lancet Neurol. *14*, 506–517.

7. Thompson, H.J., McCormick, W.C., and Kagan, S.H. (2006). Traumatic brain injury in older adults: epidemiology, outcomes, and future implications. J. Am. Geriatr. Soc. *54*, 1590–1595.

8. Edwards, G., 3rd, Moreno-Gonzalez, I., and Soto, C. (2017). Amyloid-beta and tau pathology following repetitive mild traumatic brain injury. Biochem. Biophys. Res. Commun. *483*, 1137–1142.

9. Johnson, V.E., Stewart, W., and Smith, D.H. (2010). Traumatic brain injury and amyloid-β pathology: a link to Alzheimer's disease? Nat. Rev. Neurosci. *11*, 361–370.

10. Chen, X.H., Johnson, V.E., Uryu, K., Trojanowski, J.Q., and Smith, D.H. (2009). A lack of amyloid beta plaques despite persistent accumulation of amyloid beta in axons of long-term survivors of traumatic brain injury. Brain Pathol. *19*, 214–223.

11. Ramos-Cejudo, J., Wisniewski, T., Marmar, C., Zetterberg, H., Blennow, K., de Leon, M.J., and Fossati, S. (2018). Traumatic brain injury and Alzheimer's disease: the cerebrovascular link. EBioMedicine *28*, 21–30.

12. Joshi, P., Turola, E., Ruiz, A., Bergami, A., Libera, D.D., Benussi, L., Giussani, P., Magnani, G., Comi, G., Legname, G., et al. (2014). Microglia convert aggregated amyloid-β into neurotoxic forms through the shedding of microvesicles. Cell Death Differ. *21*, 582–593.

13. Trotta, T., Panaro, M.A., Cianciulli, A., Mori, G., Di Benedetto, A., and Porro, C. (2018). Microglia-derived extracellular vesicles in Alzheimer's disease: a double-edged sword. Biochem. Pharmacol. *148*, 184–192.

14. Porro, C., Trotta, T., and Panaro, M.A. (2015). Microvesicles in the brain: biomarker, messenger or mediator? J. Neuroimmunol. *288*, 70–78.

15. Reed-Geaghan, E.G., Savage, J.C., Hise, A.G., and Landreth, G.E. (2009). CD14 and toll-like receptors 2 and 4 are required for fibrillar Abeta-stimulated microglial activation. J. Neurosci. *29*, 11982–11992.

16. Yuyama, K., and Igarashi, Y. (2017). Exosomes as carriers of Alzheimer's amyloid-ß. Front. Neurosci. *11*, 229.

17. Benilova, I., Karran, E., and De Strooper, B. (2012). The toxic Aβ oligomer and Alzheimer's disease: an emperor in need of clothes. Nat. Neurosci. *15*, 349–357.

18. Agosta, F., Dalla Libera, D., Spinelli, E.G., Finardi, A., Canu, E., Bergami, A., Bocchio Chiavetto, L., Baronio, M., Comi, G., Martino, G., et al. (2014). Myeloid microvesicles in cerebrospinal fluid are associated with myelin damage and neuronal loss in mild cognitive impairment and Alzheimer disease. Ann. Neurol. *76*, 813–825.

19. Gouwens, L.K., Ismail, M.S., Rogers, V.A., Zeller, N.T., Garrad, E.C., Amtashar, F.S., Makoni, N.J., Osborn, D.C., and Nichols, M.R. (2018). Aβ42 protofibrils interact with and are trafficked through microglial-derived microvesicles. ACS Chem. Neurosci. *9*, 1416–1425.

20. Hickman, S.E., Allison, E.K., and El Khoury, J. (2008). Microglial dysfunction and defective beta-amyloid clearance pathways in aging Alzheimer's disease mice. J. Neurosci. *28*, 8354–8360.

21. Guadagno, J., Swan, P., Shaikh, R., and Cregan, S.P. (2015). Microglia-derived IL-1β triggers p53-mediated cell cycle arrest and apoptosis in neural precursor cells. Cell Death Dis. *6*, e1779.

22. Ge, X., Yu, J., Huang, S., Yin, Z., Han, Z., Chen, F., Wang, Z., Zhang, J., and Lei, P. (2018). A novel repetitive mild traumatic brain injury mouse model for chronic traumatic encephalopathy research. J. Neurosci. Methods *308*, 162–172.

23. Huang, S., Ge, X., Yu, J., Han, Z., Yin, Z., Li, Y., Chen, F., Wang, H., Zhang, J., and Lei, P. (2018). Increased miR-124-3p in microglial exosomes following traumatic brain injury inhibits neuronal inflammation and contributes to neurite outgrowth *via* their transfer into neurons. FASEB J. *32*, 512–528.

24. Song, Y., Li, Z., He, T., Qu, M., Jiang, L., Li, W., Shi, X., Pan, J., Zhang, L., Wang, Y., et al. (2019). M2 microglia-derived exosomes protect the mouse brain from ischemia-reperfusion injury via exosomal miR-124. Theranostics *9*, 2910–2923.

25. Gordon, S. (2003). Alternative activation of macrophages. Nat. Rev. Immunol. *3*, 23–35.

26. Ransohoff, R.M. (2016). A polarizing question: do M1 and M2 microglia exist? Nat. Neurosci. *19*, 987–991.

27. Jamal, M., Ito, A., Tanaka, N., Miki, T., Takakura, A., Suzuki, S., Ameno, K., and Kinoshita, H. (2018). The role of apolipoprotein E and ethanol exposure in age-related changes in choline acetyltransferase and brain-derived neurotrophic factor expression in the mouse hippocampus. J. Mol. Neurosci. *65*, 84–92.

28. Li, L., Li, Y., Ji, X., Zhang, B., Wei, H., and Luo, Y. (2008). The effects of retinoic acid on the expression of neurogranin after experimental cerebral ischemia. Brain Res. *1226*, 234–240.

29. Peacock, W.F., 4th, Van Meter, T.E., Mirshahi, N., Ferber, K., Gerwien, R., Rao, V., Sair, H.I., Diaz-Arrastia, R., and Korley, F.K. (2017). Derivation of a three biomarker panel to improve diagnosis in patients with mild traumatic brain injury. Front. Neurol. *8*, 641.

30. Kvartsberg, H., Duits, F.H., Ingelsson, M., Andreasen, N., Öhrfelt, A., Andersson, K., Brinkmalm, G., Lannfelt, L., Minthon, L., Hansson, O., et al. (2015). Cerebrospinal fluid levels of the synaptic protein neurogranin correlates with cognitive decline in prodromal Alzheimer's disease. Alzheimers Dement. *11*, 1180–1190.

31. Wellington, H., Paterson, R.W., Portelius, E., Törnqvist, U., Magdalinou, N., Fox, N.C., Blennow, K., Schott, J.M., and Zetterberg, H. (2016). Increased CSF neurogranin concentration is specific to Alzheimer disease. Neurology *86*, 829–835.

32. Bradley-Whitman, M.A., Roberts, K.N., Abner, E.L., Scheff, S.W., Lynn, B.C., and Lovell, M.A. (2018). A novel method for the rapid detection of post-translationally modified visinin-like protein 1 in rat models of brain injury. Brain Inj. *32*, 363–380.

33. Tarawneh, R., Head, D., Allison, S., Buckles, V., Fagan, A.M., Ladenson, J.H., Morris, J.C., and Holtzman, D.M. (2015). Cerebrospinal fluid markers of neurodegeneration and rates of brain atrophy in early Alzheimer disease. JAMA Neurol. *72*, 656–665.

34. Stejskal, D., Sporova, L., Svestak, M., and Karpisek, M. (2011). Determination of serum visinin like protein-1 and its potential for the diagnosis of brain injury due to the stroke: a pilot study. Biomed. Pap. Med. Fac. Univ. Palacky Olomouc Czech Repub. *155*, 263–268.

35. McKee, A.C., Stein, T.D., Kiernan, P.T., and Alvarez, V.E. (2015). The neuropathology of chronic traumatic encephalopathy. Brain Pathol. *25*, 350–364.

36. Haass, C., and Selkoe, D.J. (2007). Soluble protein oligomers in neurodegeneration: lessons from the Alzheimer's amyloid beta-peptide. Nat. Rev. Mol. Cell Biol. *8*, 101–112.

37. Jiang, Q., Lee, C.Y., Mandrekar, S., Wilkinson, B., Cramer, P., Zelcer, N., Mann, K., Lamb, B., Willson, T.M., Collins, J.L., et al. (2008). ApoE promotes the proteolytic degradation of Abeta. Neuron *58*, 681–693.

38. Venkat, P., Cui, C., Chopp, M., Zacharek, A., Wang, F., Landschoot-Ward, J., Shen, Y., and Chen, J. (2019). MiR-126 mediates brain endothelial cell exosome treatment-induced neurorestorative effects after stroke in type 2 diabetes mellitus mice. Stroke *50*, 2865–2874.

39. Qin, Z., Wang, P.Y., Su, D.F., and Liu, X. (2016). miRNA-124 in immune system and immune disorders. Front. Immunol. *7*, 406.

40. Cunha, C., Gomes, C., Vaz, A.R., and Brites, D. (2016). Exploring new inflammatory biomarkers and pathways during LPS-induced M1 polarization. Mediators Inflamm. *2016*, 6986175.

41. Ponomarev, E.D., Veremeyko, T., Barteneva, N., Krichevsky, A.M., and Weiner, H.L. (2011). MicroRNA-124 promotes microglia quiescence and suppresses EAE by deactivating macrophages via the C/EBP-α-PU.1 pathway. Nat. Med. *17*, 64–70.

42. Yu, A., Zhang, T., Duan, H., Pan, Y., Zhang, X., Yang, G., Wang, J., Deng, Y., and Yang, Z. (2017). MiR-124 contributes to M2 polarization of microglia and confers brain inflammatory protection via the C/EBP-α pathway in intracerebral hemorrhage. Immunol. Lett. *182*, 1–11.

43. Faden, A.I., and Loane, D.J. (2015). Chronic neurodegeneration after traumatic brain injury: Alzheimer disease, chronic traumatic encephalopathy, or persistent neuroinflammation? Neurotherapeutics *12*, 143–150.

44. Chen, S., Jiang, S., Zheng, W., Tu, B., Liu, S., Ruan, H., and Fan, C. (2017). RelA/p65 inhibition prevents tendon adhesion by modulating inflammation, cell proliferation, and apoptosis. Cell Death Dis. *8*, e2710.

45. Dabrowska, S., Andrzejewska, A., Lukomska, B., and Janowski, M. (2019). Neuroinflammation as a target for treatment of stroke using mesenchymal stem cells and extracellular vesicles. J. Neuroinflammation *16*, 178.

46. Weisgraber, K.H. (1994). Apolipoprotein E: structure-function relationships. Adv. Protein Chem. *45*, 249–302.

47. Chauhan, N.B. (2014). Chronic neurodegenerative consequences of traumatic brain injury. Restor. Neurol. Neurosci. *32*, 337–365.

48. Laskowitz, D.T., Goel, S., Bennett, E.R., and Matthew, W.D. (1997). Apolipoprotein E suppresses glial cell secretion of TNF alpha. J. Neuroimmunol. *76*, 70–74.

49. Laskowitz, D.T., Wang, H., Chen, T., Lubkin, D.T., Cantillana, V., Tu, T.M., Kernagis, D., Zhou, G., Macy, G., Kolls, B.J., and Dawson, H.N. (2017). Neuroprotective pentapeptide CN-105 is associated with reduced sterile inflammation and improved functional outcomes in a traumatic brain injury murine model. Sci. Rep. *7*, 46461.

50. Namjoshi, D.R., Martin, G., Donkin, J., Wilkinson, A., Stukas, S., Fan, J., Carr, M., Tabarestani, S., Wuerth, K., Hancock, R.E., and Wellington, C.L. (2013). The liver X receptor agonist GW3965 improves recovery from mild repetitive traumatic brain injury in mice partly through apolipoprotein E. PLoS ONE *8*, e53529.

51. Hooper, C., Sainz-Fuertes, R., Lynham, S., Hye, A., Killick, R., Warley, A., Bolondi, C., Pocock, J., and Lovestone, S. (2012). Wnt3a induces exosome secretion from primary cultured rat microglia. BMC Neurosci. *13*, 144.

52. Asai, H., Ikezu, S., Tsunoda, S., Medalla, M., Luebke, J., Haydar, T., Wolozin, B., Butovsky, O., Kügler, S., and Ikezu, T. (2015). Depletion of microglia and inhibition of exosome synthesis halt tau propagation. Nat. Neurosci. *18*, 1584–1593.

53. Glebov, K., Löchner, M., Jabs, R., Lau, T., Merkel, O., Schloss, P., Steinhäuser, C., and Walter, J. (2015). Serotonin stimulates secretion of exosomes from microglia cells. Glia *63*, 626–634.

54. Cirrito, J.R., Disabato, B.M., Restivo, J.L., Verges, D.K., Goebel, W.D., Sathyan, A., Hayreh, D., D'Angelo, G., Benzinger, T., Yoon, H., et al. (2011). Serotonin signaling is associated with lower amyloid-β levels and plaques in transgenic mice and humans. Proc. Natl. Acad. Sci. USA *108*, 14968–14973.

55. Zhang, Y., Chopp, M., Meng, Y., Katakowski, M., Xin, H., Mahmood, A., and Xiong, Y. (2015). Effect of exosomes derived from multipluripotent mesenchymal stromal cells on functional recovery and neurovascular plasticity in rats after traumatic brain injury. J. Neurosurg. *122*, 856–867.

56. Zhang, Y., Chopp, M., Zhang, Z.G., Katakowski, M., Xin, H., Qu, C., Ali, M., Mahmood, A., and Xiong, Y. (2017). Systemic administration of cell-free exosomes generated by human bone marrow derived mesenchymal stem cells cultured under 2D and 3D conditions improves functional recovery in rats after traumatic brain injury. Neurochem. Int. *111*, 69–81.

57. Kim, D.K., Nishida, H., An, S.Y., Shetty, A.K., Bartosh, T.J., and Prockop, D.J. (2016). Chromatographically isolated CD63+CD81+ extracellular vesicles from mesenchymal stromal cells rescue cognitive impairments after TBI. Proc. Natl. Acad. Sci. USA *113*, 170–175.

58. Goetzl, E.J., Schwartz, J.B., Abner, E.L., Jicha, G.A., and Kapogiannis, D. (2018). High complement levels in astrocyte-derived exosomes of Alzheimer disease. Ann. Neurol. *83*, 544–552.

59. Goetzl, E.J., Kapogiannis, D., Schwartz, J.B., Lobach, I.V., Goetzl, L., Abner, E.L., Jicha, G.A., Karydas, A.M., Boxer, A., and Miller, B.L. (2016). Decreased synaptic proteins in neuronal exosomes of frontotemporal dementia and Alzheimer's disease. FASEB J. *30*, 4141–4148.

60. Han, Z., Ge, X., Tan, J., Chen, F., Gao, H., Lei, P., and Zhang, J. (2015). Establishment of lipofection protocol for efficient miR-21 transfection into cortical neurons in vitro. DNA Cell Biol. *34*, 703–709.

61. Ge, X., Huang, S., Gao, H., Han, Z., Chen, F., Zhang, S., Wang, Z., Kang, C., Jiang, R., Yue, S., et al. (2016). miR-21-5p alleviates leakage of injured brain microvascular endothelial barrier in vitro through suppressing inflammation and apoptosis. Brain Res. *1650*, 31–40.

62. Zhang, Y., Chopp, M., Liu, X.S., Katakowski, M., Wang, X., Tian, X., Wu, D., and Zhang, Z.G. (2017). Exosomes derived from mesenchymal stromal cells promote axonal growth of cortical neurons. Mol. Neurobiol. *54*, 2659–2673.

63. Ge, X., Li, W., Huang, S., Yin, Z., Yang, M., Han, Z., Chen, F., Wang, H., Lei, P., and Zhang, J. (2019). Increased miR-21-3p in injured brain microvascular endothelial cells after traumatic brain injury aggravates blood-brain barrier damage by promoting cellular apoptosis and inflammation through targeting MAT2B. J. Neurotrauma *36*, 1291–1305.

64. Ge, X., Han, Z., Chen, F., Wang, H., Zhang, B., Jiang, R., Lei, P., and Zhang, J. (2015). MiR-21 alleviates secondary blood-brain barrier damage after traumatic brain injury in rats. Brain Res. *1603*, 150–157.

65. Ge, X.T., Lei, P., Wang, H.C., Zhang, A.L., Han, Z.L., Chen, X., Li, S.H., Jiang, R.C., Kang, C.S., and Zhang, J.N. (2014). miR-21 improves the neurological outcome after traumatic brain injury in rats. Sci. Rep. *4*, 6718.

66. Leger, M., Quiedeville, A., Bouet, V., Haelewyn, B., Boulouard, M., Schumann-Bard, P., and Freret, T. (2013). Object recognition test in mice. Nat. Protoc. *8*, 2531–2537.

67. Zhang, Y., Kim, M.S., Jia, B., Yan, J., Zuniga-Hertz, J.P., Han, C., and Cai, D. (2017). Hypothalamic stem cells control ageing speed partly through exosomal miRNAs. Nature *548*, 52–57.

YMTHE, Volume *28*

Supplemental Information

Increased Microglial Exosomal miR-124-3p Alleviates Neurodegeneration and Improves Cognitive Outcome after rmTBI

Xintong Ge, Mengtian Guo, Tianpeng Hu, Wenzhu Li, Shan Huang, Zhenyu Yin, Ying Li, Fanglian Chen, Luoyun Zhu, Chunsheng Kang, Rongcai Jiang, Ping Lei, and Jianning Zhang

Supplemental Figures

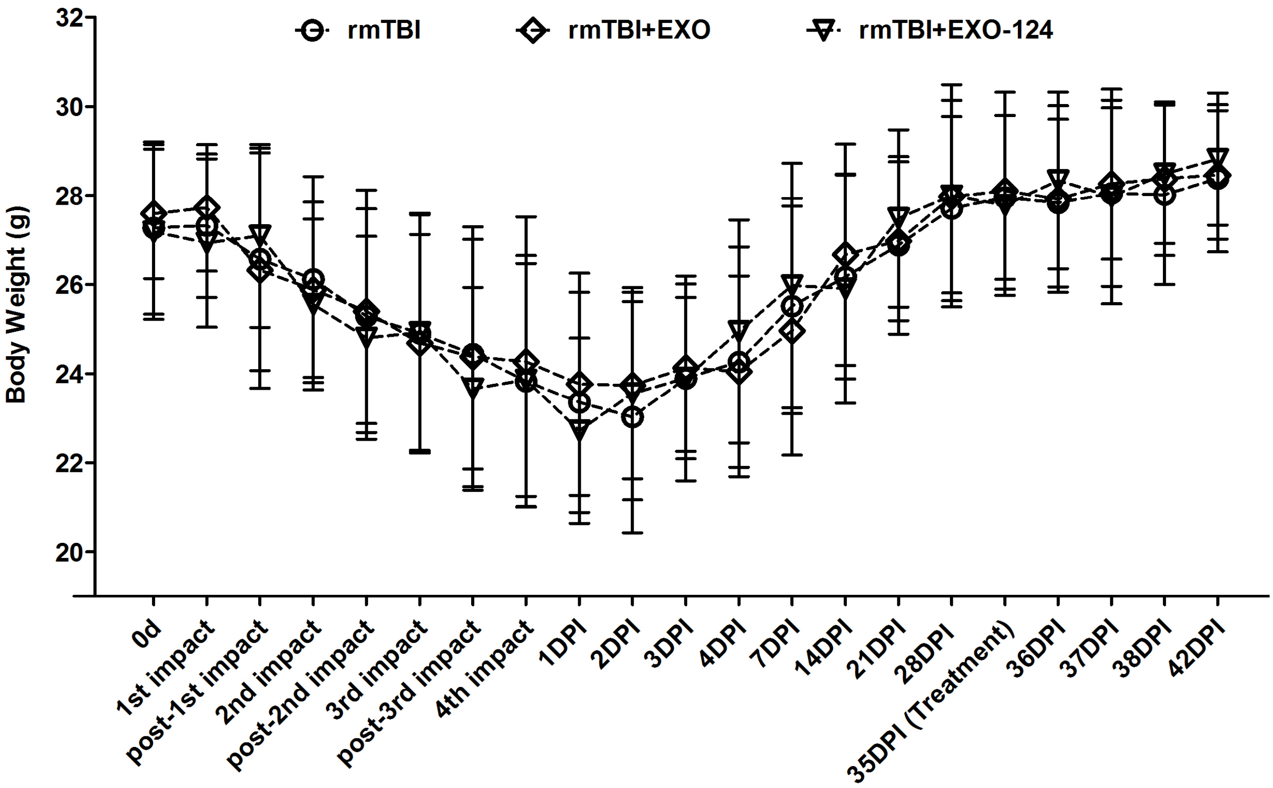

Figure S1. Changes on the body weight of rmTBI mice during the impacting procedure and after injury. The rmTBI mice did loss body weight during the repetitive impacting procedure, but their body weights gradually recovered before receiving treatment at 35 DPI. In addition, no difference was observed among the treatment groups. Abbreviation in the figure: DPI: days post-injury.

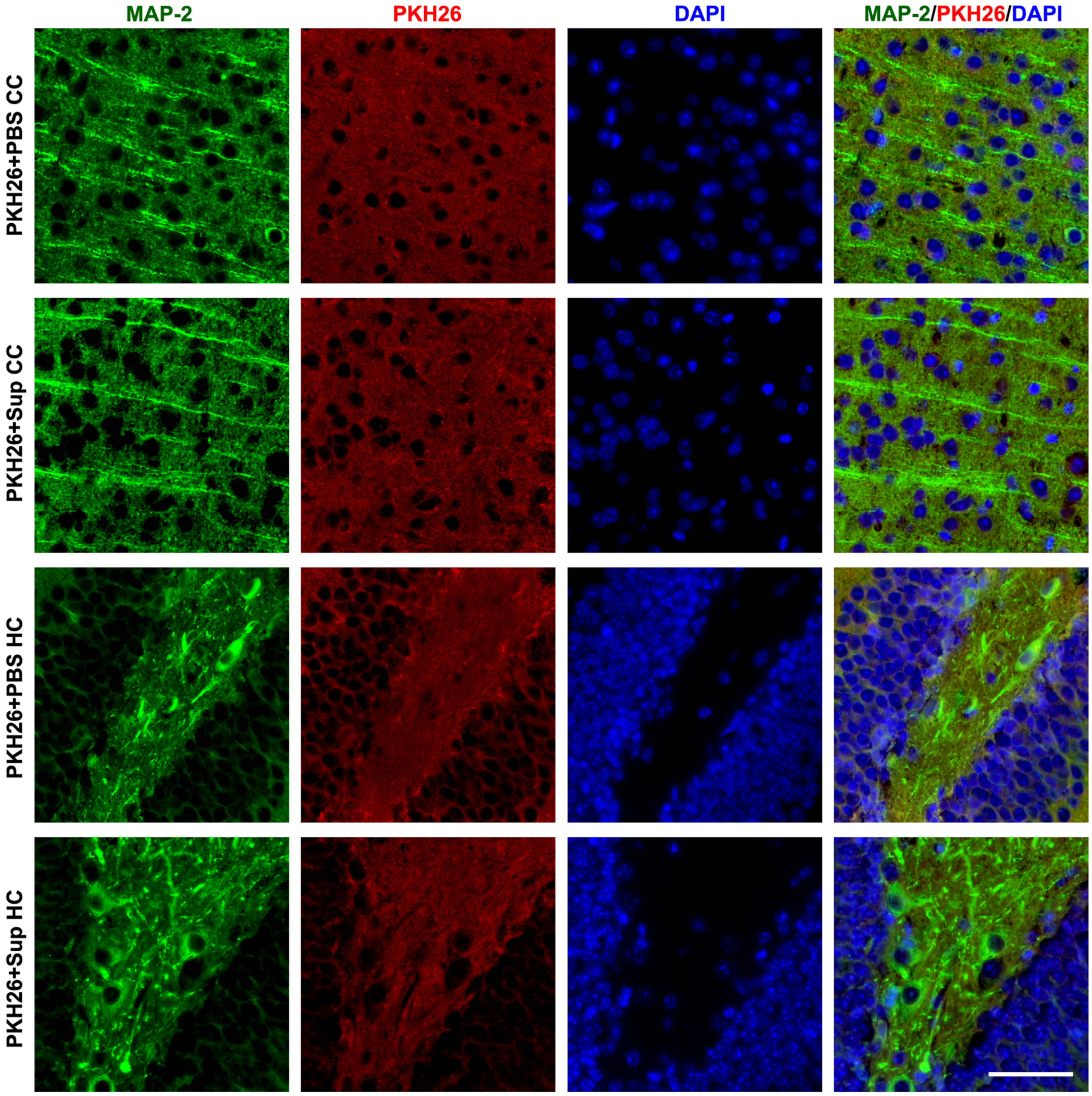

Figure S2. The negative control images of immunofluorescence staining for PKH26 labeled-microglial exosomes in rmTBI mice brain. PKH26 added to PBS and the enriched supernatant (the culture medium of microglia after ultracentrifugation) were injected into rmTBI mice as negative control. Note that PKH26 could be barely observed in the CC and the HC of rmTBI mice at 3 DPI. Scale bars: 50 μm. Abbreviations in the figure: DPI: days post-injury, CC: cerebral cortex, HC: hippocampus.

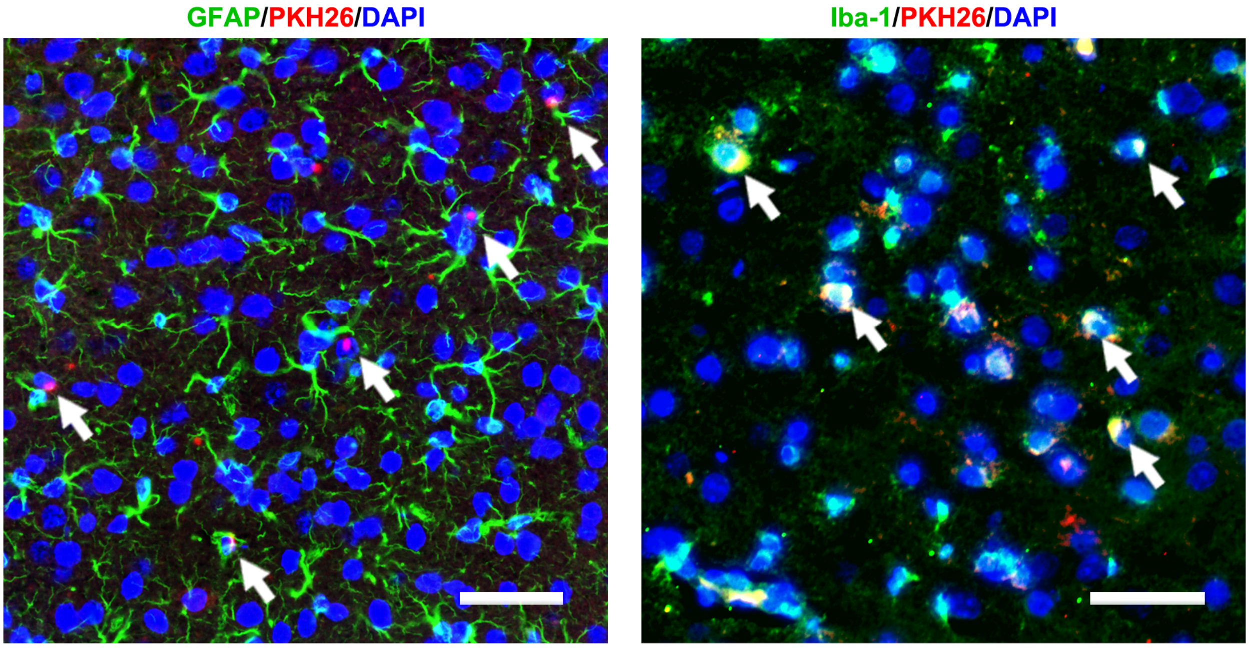

Figure S3. Immunofluorescence staining of astrocytes and microglia in the cerebral cortex of rmTBI mice, taking up exogenous administrated microglial exosomes. The intravenously injected exosomes could be taken up by astrocytes (marked by co-expression of GFAP and PKH26) and microglia (marked by co-expression of Iba-1 and PKH26) at 42 DPI. The double immunostained cells are indicated by white arrows. Note that the number of different types of co-localized neural cells (neurons, astrocytes and microglia) in the pictures has no comparative significance. There is no difference in the number of neurons and astrocytes that co-localized with PKH26 labeled exosomes. Scale bars: 50 μm. Abbreviation in the figure: DPI: days post-injury.
